# LncRNA PVT1 links estrogen receptor alpha and the polycomb repressive complex 2 in suppression of pro-apoptotic genes in hormone-responsive breast cancer

**DOI:** 10.1038/s41419-025-07423-4

**Published:** 2025-02-08

**Authors:** Viola Melone, Domenico Palumbo, Luigi Palo, Noemi Brusco, Annamaria Salvati, Antonietta Tarallo, Giorgio Giurato, Francesca Rizzo, Giovanni Nassa, Alessandro Weisz, Roberta Tarallo

**Affiliations:** 1 Laboratory of Molecular Medicine and Genomics, Department of Medicine, Surgery and Dentistry “Scuola Medica Salernitana”, University of Salerno, 84081 Baronissi, SA Italy; 2 Genome Research Center for Health, 84081 Baronissi, SA Italy; 3 Department of Translational Medical Sciences, Federico II University, 80131 Naples, Italy; 4 Telethon Institute of Genetics and Medicine, 80078 Pozzuoli, Italy; 5 Medical Genomics Program and Division of Oncology, AOU ‘S. Giovanni di Dio e Ruggi d’Aragona’ University of Salerno, and Rete Oncologica Campana, 84131 Salerno, Italy

**Keywords:** Cancer genomics, Long non-coding RNAs, Prognostic markers, Breast cancer

## Abstract

RNA-based therapeutics highlighted novel approaches to target either coding or noncoding molecules for multiple diseases treatment. In breast cancer (BC), a multitude of deregulated long noncoding RNAs (lncRNAs) have been identified as potential therapeutic targets also in the context of antiestrogen resistance, and the RNA binding activity of the estrogen receptor α (ERα) points additional potential candidates to interfere with estrogenic signaling. A set of lncRNAs was selected among ERα-associated RNAs in BC cell nuclei due to their roles in processes such as transcriptional regulation and epigenetic chromatin modifications. Native immunoprecipitation of nuclear ERα-interacting RNAs coupled to NGS (RIP-Seq) was performed in MCF-7 cells, leading to the identification of essential lncRNAs interacting with the receptor in multi-molecular regulatory complexes. Among these, PVT1, FGD5-AS1 and EPB41L4A-AS1 were selected for further investigation. Functional assays and transcriptome analysis following lncRNA knock-down indicated PVT1 as the master modulator of some of the most relevant BC hallmarks, such as cell proliferation, apoptosis, migration and response to hypoxia. In addition, targeted experiments identified PVT1 as a key factor in the composition of PRC2-ERα network involved in downregulation of tumor suppressor genes, including BTG2.

## Introduction

The increasing knowledge of the broad spectrum of functional roles exerted by different classes of RNAs allowed, in recent years, the advancement of RNA therapeutics from a hypothetical concept to clinical reality, thus providing a novel way to target coding as well as noncoding RNAs (ncRNAs) for the customized treatment of multiple diseases. Indeed, deregulated gene expression may be controlled, either permanently or transiently, by directly targeting specific RNAs or proteins with the aid of small molecules endowed with therapeutic action. Therefore, RNA-binding proteins (RBPs) and their connected molecules play crucial roles in this context [1]. RBPs form ribonucleoprotein complexes that specifically modulate gene expression through alternative splicing, RNA decay, translocation or translation [2]. Moreover, their aberrant activity was described to contribute to cancerous transformation and/or resistance to therapies [3]. In this view, the disruption of RNA–protein networks represents a promising avenue for cancer therapeutics, especially in the absence of “druggable” molecules, or to counteract the occurrence of drug resistance [1].

In breast cancer (BC), the discovery that the estrogen receptor α (ERα), the main hallmark of luminal-like hormone-responsive BC subtype, acts as a non-canonical RBP, brought out a new oncogenic signature under the control of this factor [4]. It has been well established that, upon estrogen stimulation, ERα is recruited, together with a host of transcriptional coregulators, onto specific target sites within the chromatin for modulation of target genes expression. Indeed, ERα can interact with epigenetic readers, writers and erasers within multimolecular complexes able to regulate gene expression in a combinatorial manner [5, 6]. More recent evidences demonstrated also the ability of ERα to bind several RNA species, among which a crucial functional role is likely to be exerted by ncRNAs [4].

Furthermore, a multitude of deregulated ncRNAs has been described as novel potential “druggable” targets for BC treatment [7, 8]. Among these, the role of long ncRNAs (lncRNAs) have been investigated for their involvement in both physiological and pathological processes [9–11]. These are RNA molecules more than 200 nucleotides long that are transcribed by RNA polymerase II, 5’ capped, polyadenylated and, in most cases, lack an open reading frame (ORF) and therefore protein-coding ability [11, 12]. Recently, lncRNAs received growing interest for their involvement in multiple cellular processes and for their functional roles in epigenetics, transcriptional and post-transcriptional events, making them attractive target candidates for improvement of cancer treatment efficiency [13, 14].

The blockade of the estrogenic signaling through ERα inhibition using selective estrogen receptor modulators (SERMs), selective estrogen receptor downregulators (SERDs) or aromatase inhibitors (AIs) represented, for many years, the first line approach for hormone-based therapy in patients suffering with ERα-positive BC [15]. The main problem, occurring in ~30% of the cases, is the acquisition of resistance to hormone therapies, nowadays representing the main cause of death for these patients [16]. Despite recent advancements in treatments based, for example, on new targeted and chemotherapies, BC remains a significant threat for women’s health, and this points to the urgent need to find novel targets to overcome this hindrance [2, 7].

The role of ERα as RBP is still not well defined and understood, although representing a new central piece of the puzzle. In this study, we investigated whether the receptor would be enrolled in modulating the expression of target genes, important for tumor sustainment and neoplastic transformation, through the association, within the nuclear compartment, with specific lncRNAs. Firstly, ERα-interacting lncRNAs were identified by using RNA-immunoprecipitation coupled to sequencing (RIP-Seq), then, once identified the most informative interacting RNAs, we evaluated the functional effects of their silencing on cell proliferation, induction of apoptotic cell death and modulation of the estrogenic signaling after RNA knock-down with antisense-oligonucleotides (ASOs). Out of three lncRNAs selected among those previously demonstrated to be essential for BC cell growth [17], PVT1 emerged as the most significantly associated with BC prognosis, since its overexpression correlated with worse overall survival in TCGA and TARGET Pan Cancer patients. Moreover, it emerged as a key component of the estrogenic signaling acting as a bridge factor between Polycomb Repressive Complex 2 (PRC2) activity and ERα transcriptional modulation. In particular, the experimental results identified PVT1 as a core molecule in the composition of a transcriptional repressive complex allowing the functional cooperation of PRC2 and ERα through PVT1-mediated association of EZH2 and the receptor with the transcription unit of tumor suppressive genes such as BTG2 (BTG family member 2/NGF-inducible anti-proliferative protein PC3). Targeting PVT1 by means of ASO-based silencing may thus represent an alternative way to clinically interfere with estrogenic signaling and activate the apoptotic cascade in BC.

## Materials and methods

For detailed methods, see Supplementary Material.

### RNA immunoprecipitation

For ERα-associated RNA immunoprecipitation (RIP), 7 μg of anti-ERα or anti-IgG Isotype Control were conjugated overnight at 4 °C with 100 μl of Dynabeads M-280 Sheep AntiRabbit IgG (Thermo Fisher). Cells were washed twice with cold PBS supplemented with 0.1% of EDTA (500 mM), harvested by scraping and collected in a tube. After a centrifugation at 3000 rpm for 5 min at 4 °C, the nuclear fraction was extracted by resuspending the pellet in nuclear isolation Buffer (NIB) (1.28 M Sucrose, 40 mM Tris-HCl pH 7.5, 20 mM MgCl2 and 4% Triton X-100) supplemented with 100 U/ml RNAse inhibitor (RiboLock RNase Inhibitor, Invitrogen) and proteinase inhibitors (1 mM PMSF and 1× PIC). Samples were incubated on ice for 20 min and then centrifuged at 2500 × *g* at 4 °C for 15 min. Once removed the supernatant, nuclear pellets were resuspended in 400 μl of RIP Buffer (150 mM KCl, 25 mM Tris pH 7.4, 5 mM EDTA, 0.5 mM DTT e 0.5% NP40) supplemented with 100 U/ml RNAse and proteinase (1 mM PMSF and 1x PIC) inhibitors. Cell nuclei were sonicated for 10 cycles (15” ON and 15” OFF) using Bioruptor (Diagenode, Denville, New Jersey, USA) and subsequently centrifuged at 13,000 rpm for 10 min at 4 °C. The resulting supernatant represented the nuclear protein extract whose concentration was measured with Bradford assay. Then, 2.5 mg of nuclear extract was incubated at 4 °C for 2 h with the conjugated beads/antibodies. After the binding, beads were washed 3 times with RIP Buffer in rotation for 5 min at 4 °C, and twice quickly. Once discarded all the supernatant, 1 ml of TRIzol^TM^ (Life Technologies, Thermo Fisher) was added directly to the beads and RNA extraction was performed according to the manufacturer’s guidelines.

### RNA sequencing

Libraries preparation for transcriptome profiling was performed by using the TruSeq Stranded Total RNA Library Prep Gold (Cat. 20020599, Illumina, San Diego, California, USA) for RIP-Seq and silencing experiments, while the lllumina Stranded Total RNA prep Ligation with Ribo-Zero Plus kit (Cat. 20040529, Illumina) was employed for Nascent-Seq, according to manufacturers’ guidelines. RIP-Seq libraries were sequenced on NextSeq 500 (Illumina) using 2 × 75 bp paired end mode. Total RNA-Seq and Nascent-RNA Seq libraries were sequenced on Novaseq 6000 platform (Illumina) using 2 × 100 bp paired end mode.

### Chorioallantoic membrane (CAM) assay

CAM assay was performed as previously described by Bianco et al. [18] with minor modifications. Briefly, fertilized chicken eggs were equilibrated at 37 °C in a humidified incubator from the 1st until the 8th day of gestation. Then an artificial air sac was designed by piercing the eggshell with a needle into the air sac and near the allantoic vein. Subsequently, a vacuum was applied to the air sac hole to detach CAM from the eggshell and a perforation of 1 cm^2^, near the allantoic vein, was performed to expose the CAM. MCF-7 cells, 6 h post ASO transfection, were inoculated at 5 × 10^6^ cells/each CAM. In details, transfected cells were detached from the culture dish, counted and suspended in 40 μl of a 1:1 mixture composed of growth medium and BME (Cat. #3533-005-02, R&D Systems, Minneapolis, USA). Cells were inoculated in an 8 mm sterile Teflon ring placed on the membrane to prevent cells spreading. Embryos were incubated for 4 days after which tumors were excised from the site of inoculation. Surface measurements were performed by averaging the areas (width*width) of each tumor.

### Data analysis

Nascent, Total RNA-Seq and RIP-Seq data analysis was performed as follows: Fastq were generated from bcl files using bcl2fastq (Illumina v2.20.0.422), while the quality check was assessed using FastQC (v0.11.9) [19]. The adapters were removed using cutadapt (v 3.3) [20] and the resulted fastq were aligned on human genome (hg38) using STAR [21] (v 2.7.8a) with assembly of GENCODE v37 as GTF file. The raw counts were generated using featurecounts (v2.0.1) [22]. Differential expression analysis and the normalized counts were produced using DESeq2 (v 1.38.3) on R (4.2.2). Transcripts were considered differentially expressed if they showed |FC| ≥ 1.5 and adjusted *p*-value ≤ 0.05 or enriched if they showed |FC| > 1. Volcano plots were generated using EnhancedVolcano (v 1.16.0) while Gene Ontology circos with GOplot (v 1.0.2). All sequencing data are available in ArrayExpress database with the following accession numbers: E-MTAB-14147, E-MTAB-14148, E-MTAB-14149.

Furthermore, boxplots with TGCA data were produced on GEPIA2 [23] while TCGA and TARGET Pan Cancer BRCA data available on UCSC Xena tool were used for the survival plot [24]. All the barplots, the heatmaps and the related statistics were made using GraphPad Prism version 8.0.0 for Windows, GraphPad Software, Boston, Massachusetts USA, www.graphpad.com. All other plots and statistics were produced on R with an alpha value set for *p* < 0.05. Predictions of lncRNAs interaction with DNA were calculated with the online version of LongTarget (www.gaemons.net) [25] using the default parameters and the entire hg38 for genome scale prediction. Sequence Searcher [26] was used to identify the presence of ERE or imperfect ERE (allowing a maximum of 2 mismatches) as explained by Driscoll et al. [27]. Chip and CUT&Tag data, for EZH2 and H3K27me3 respectively, were downloaded from GEODataset (GSE201262) produced by Tian et al. [28], while ERα Clip-Seq was obtained from supplementary material of Xu et al. [4] and converted to hg38 using liftOver from UCSC [29]. CHIA-PET long-range chromatin interactions data was downloaded from GEODataset (GSE176821). ERα Chip-Seq was re-analyzed from data previously published by Nassa et al. [30] and converted to hg38 using liftOver from UCSC.

## Results

### Identification of ERα-associated RNAs in BC cell nuclei

**Fig. 1:**
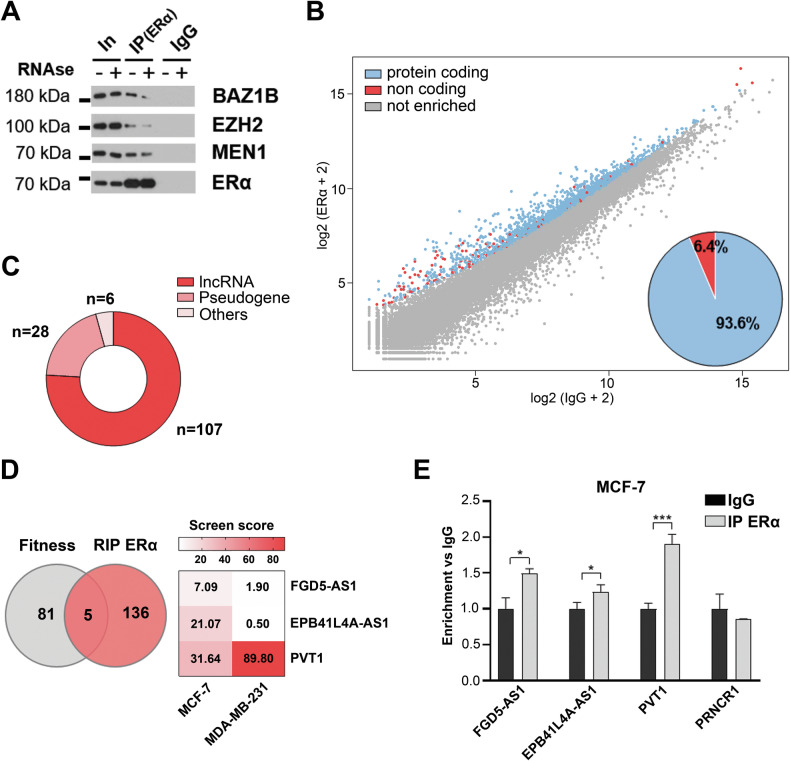
ERα interacts with several RNAs in MCF-7 BC cell nuclei. **A** WB showing BAZ1B, EZH2 and MEN1 enrichment following ERα immunoprecipitation with or without RNase treatment in MCF-7 nuclear extracts. IgG was used as negative control. **B** Scatter plot (left) showing enriched RNAs (log2 + 2) identified through ERα nuclear RIP-Seq. IgG was used as negative control. Pie chart (right) represents the enriched RNAs categories (protein coding in blue and non-coding in red). **C** Donut chart showing non-coding categories with different red shades. **D** Venn diagram (left) showing MCF-7 fitness lncRNAs among those interacting with ERα. Heatmap (right) showing screen score values of the three lncRNAs FGD5-AS1, EPB41L4A-AS1 and PVT1 selected for our investigation. Each row represents one lncRNA while the columns represent values observed in MCF-7 (left) and in ERα-negative MDA-MB-231 (right). Scale bar (up) represents the screen score (genes are considered fitness starting from a threshold screen score of 7). **E** RIP coupled to RT-qPCR validating the enrichment of ERα-associated lncRNAs in MCF-7 cell nuclei using IgG as negative control. lncRNA PRNCR1, selected among not enriched molecules, was used as negative control. The results showed are the mean ± SD of triplicate values. Asterisks indicate statistically significant differences using unpaired t-test (*p < 0.05 and ***p < 0.005).

Previous work from our group, by applying Tandem Affinity Purification (TAP) coupled to mass spectrometry (MS) followed by in vitro RNAse digestion, demonstrated quantitative changes in ERα association with a large subset of its nuclear interactors in MCF-7 BC cell nuclei [31]. These ERα molecular partners include enzymes and transcription regulators mainly demonstrated to be part of chromatin-associated multiprotein complexes required for ERα activity [31]. Among others, we noticed a reduction of ERα co-immunoprecipitation with epigenetic modulator proteins such as the Bromodomain Adjacent To Zinc Finger Domain 1B (BAZ1B), the scaffold protein menin 1 (MEN1) and the Enhancer Of Zeste 2 Polycomb Repressive Complex 2 Subunit (EZH2), previously identified to be involved with ERα in controlling key BC hallmarks (Fig. 1A) [32, 33]. This strongly suggested the potential involvement of RNA(s) in ERα-associated nuclear multimolecular complexes assembly, which may act as functional mediators of the receptor transcriptional activity in the modulation of specific target genes expression.

To investigate this hypothesis, a native ERα immunoprecipitation coupled to RNA sequencing (RIP-Seq) was performed in nuclear extracts from exponentially growing MCF-7 cells, leading to the identification of 2212 ERα-interacting RNAs (Fig. 1B, Supplementary Table S1). Among these, 2071 (93.6%) are protein coding while 141 (6.4%) are noncoding RNAs (Fig. 1B) and in them, lncRNAs (107) were selected for our investigation (Fig. 1C).

Considering the growing interest in lncRNAs in cancer biology, we directed our focus on those found to be essential for BC growth and/or survival (defined as fitness lncRNAs) by Liu et al. [17], that identified, through CRISPRi screening, fitness lncRNAs in multiple tumor cell lines including some of the more representative BC ones. The comparison between this dataset and the generated list of ERα-interacting ncRNAs identified five of them as essential in ERα-positive BC cells (Fig. 1D, Supplementary Table S2). Among these, FGD5-AS1, EPB41L4A-AS1 and PVT1 (Fig. 1D) were selected for further investigation, based on published literature, fitness score and the predicted biological meaning of their functional cooperation with ERα [17, 34–38]. The interaction between ERα and these three lncRNAs was confirmed in MCF-7 cells by RIP coupled to RT-qPCR using as negative control the lncRNA PRNCR1, which was not found here to bind ERα (Fig. 1E, Supplementary Table S1). This result was validated also with a different primers set and in both MCF-7 and T-47D cells (Supplementary Fig. S1A and B).

### Functional characterization of ERα-interacting lncRNAs

**Fig. 2:**
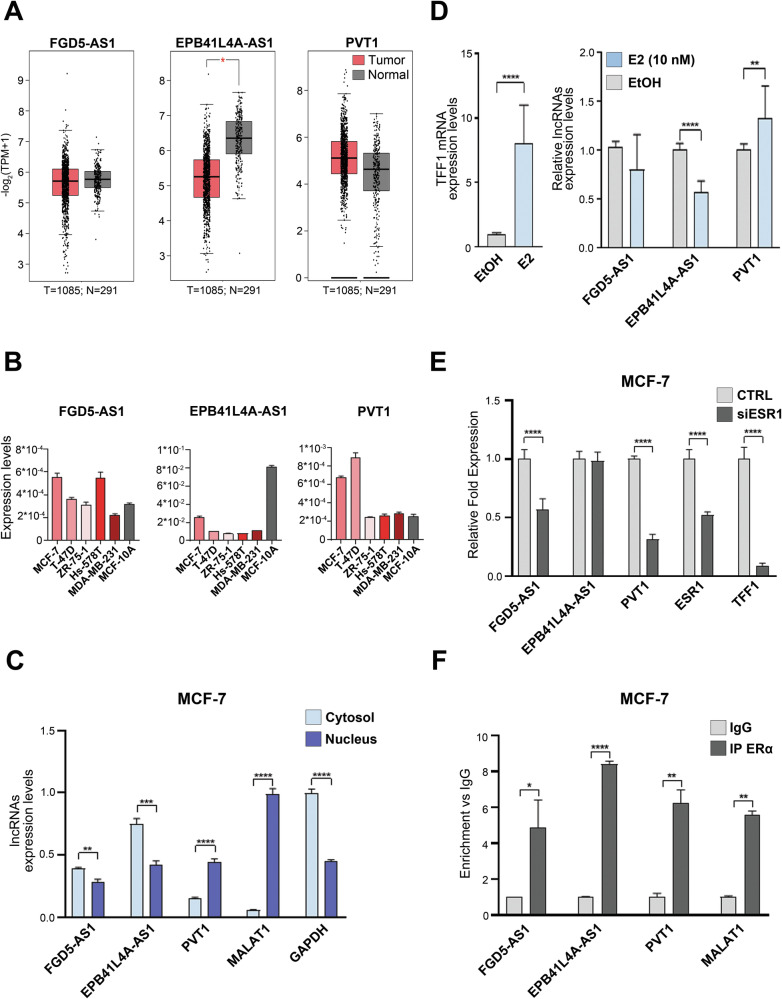
Characterization of FGD5-AS1, EPB41L4A-AS1 and PVT1 as ERα-interacting lncRNAs. **A** Box Plots showing lncRNAs expression levels (-log2(TPM + 1)) comparing tumor (red) and normal (gray) tissues from TCGA data by using GEPIA2 tool. The number of the tissues considered in the analysis is listed below each comparison. Asterisk indicates statistically significant differences using One-Way ANOVA (*p < 0.05). **B** Bar plots showing the expression levels of the selected lncRNAs in ERα-positive (MCF-7, T-47D and ZR-75-1), ERα-negative (Hs-578T, MDA-MB-231) BC cell lines an in the mammary epithelial cell line MCF-10A. RT-qPCR results shown are the mean ± SD of triplicate values using as housekeeping internal control RPLP0. **C** Bar plots showing RT-qPCR results of fractionated (cytosol and nucleus) lncRNAs distribution relative to total RNAs. MALAT1 and GAPDH were employed as housekeeping controls for nucleus and cytosol respectively. The results showed are the mean ± SD of triplicate values. Asterisks indicate statistically significant differences using unpaired t-test (**p < 0.01, ***p < 0.005 and ****p < 0.001). **D** Bar plots showing the expression levels (measured with RT-qPCR) of TFF1 mRNA (left) and of the three lncRNAs (right) following 24 h of mitogenic E2 stimulation in hormone-deprived MCF-7. RPLP0 was used as housekeeping. The results showed are the mean ± SD of three independent experiments. Asterisks indicate statistically significant differences using unpaired t-test (**p < 0.01 and ****p < 0.001). **E** Bar plots showing RT-qPCR results of relative fold expression levels of the indicated transcripts following ESR1 knock-down. ESR1 and TFF1 mRNA levels were analyzed as experimental controls. Data were analyzed with respect to the scramble (Silencer Select Negative Control: CTRL). The results showed are the mean ± SD of three independent experiments. Asterisks indicate statistically significant differences using unpaired t-test (****p < 0.001). **F** Chromatin associated RNA immunoprecipitation (CARIP) coupled to RT-qPCR showing the enrichment of ERα-associated lncRNAs on MCF-7 BC cell chromatin. IgG was used as negative control. lncRNA MALAT1 was used as positive experimental control. The results showed are the mean ± SD of triplicate values. Asterisks indicate statistically significant differences using unpaired t-test (*p < 0.05, **p < 0.01 and ****p < 0.001).

The expression levels of the selected lncRNAs were analyzed in BC RNA-Seq data from TCGA (Fig. 2A) and measured by RT-qPCR in ERα + BC cell lines (Fig. 2B). Results showed that FGD5-AS1 expression level is not significantly different in normal vs cancer tissues and in luminal-like (MCF-7, T-47D and ZR-75-1), triple negative (MDA-MB-231 and Hs-578T) BC and immortalized epithelial (MCF-10A) breast cell lines. On the contrary, EPB41L4A-AS1, previously described as a tumor suppressor [34], resulted downregulated in tumor tissues and in BC cells, compared to normal ones, while PVT1, previously described as a pan-oncogene [35–37], resulted overexpressed in both. Furthermore, in native conditions, the three RNAs displayed a different localization within the cell compartments. Specifically, EPB41L4A-AS1 and FGD5-AS1 appear more abundant in the cytoplasm whereas PVT1 prevails in the nuclear compartment (Fig. 2C).

In order to investigate their relationship with the estrogenic signaling, lncRNAs expression levels were evaluated in hormone-deprived MCF-7 cells stimulated with a mitogenic dose of estradiol (E2 10 nM) for 24 h (Fig. 2D). Interestingly, the results obtained showed a significant decrease of EPB41L4A-AS1 and an increase in PVT1 expression level upon hormonal treatment, in line with the presence of ER-binding motifs (EREs) within its transcription unit (Fig. 2D, Supplementary Fig. S2A). To further confirm that the observed modulation might be linked to ERα, the expression levels of the three lncRNAs were evaluated in MCF-7 cells following siRNA-mediated ERα gene (ESR1) knock-down. Results showed that ERα kd resulted in a decrease of both PVT1 and FGD5-AS1 levels. On the other hand, EPB41L4A-AS1 expression was unaffected by receptor downregulation, indicating that this lncRNA, although responding to estrogen stimulation, does not appear to be a direct ER target (Fig. 2E).

Chromatin associated ERα-RNA immunoprecipitation (CARIP) coupled to RT-qPCR, confirmed an enrichment of the three molecules investigated (Fig. 2F). This indicated that, although in the presence of a different nuclear concentration among them, since PVT1 results highly expressed in this compartment compared to the others, all three lncRNAs result associated with chromatin, suggesting that all exert a functional role in the genome of these BC cells. Furthermore, given the knowledge that different lncRNAs are likely to act together by assembling in multi-modular complexes for gene expression modulation [14], we first pursued to identify putative lncRNA-DNA binding motifs within MCF-7 cells genome and then compared common sites with ERα binding sites previously identified in the same experimental conditions [31]. This allowed the identification of a set of commonly occupied genes, whose expression was predicted to be regulated by a complex involving both ERα and these lncRNAs (Supplementary Fig. S2B, Supplementary Table S3), thus suggesting an involvement of these molecules in estrogen-responsive regulation of gene expression in luminal-like cells.

**Fig. 3:**
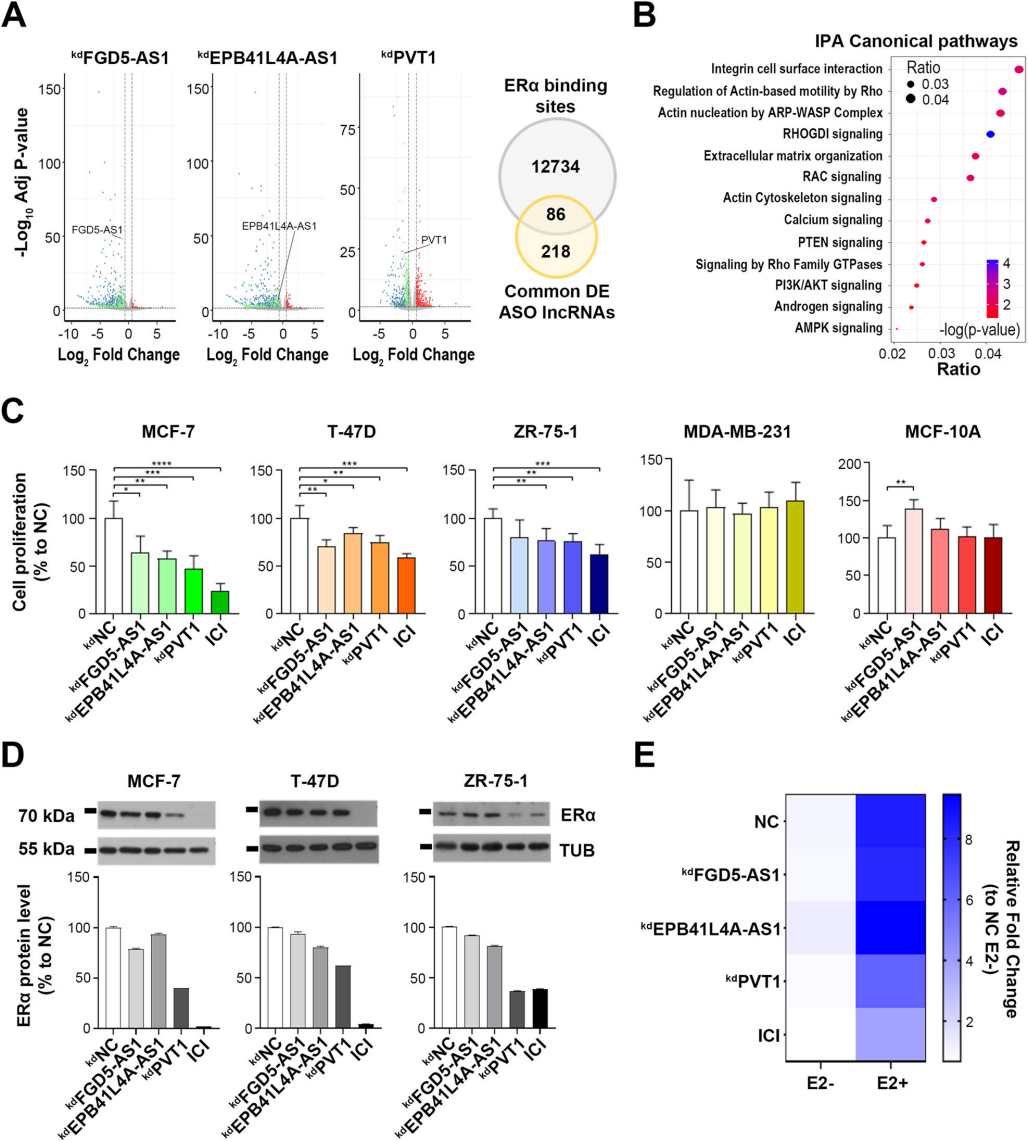
Functional portrait of FGD5-AS1, EPB41L4A-AS1 and PVT1 following ASO-mediated silencing. **A** Volcano Plots (left) displaying differentially expressed genes (green down, red up and blue common among all comparisons) identified following 72 h of ASO-mediated lncRNAs silencing. Gray dots represent not statistically significant genes (−1.5 ≥ Fold change ≤ 1.5). The Venn diagram (right) shows the intersection between ERα binding sites [30] and the common differentially expressed genes identified after lncRNAs silencing (hypergeometric test *p*-value < 1.81*10^−3^). **B** Dot plot of the most significant IPA canonical pathways involving common differentially expressed genes identified after silencing. Dot color ranges from red to blue depending on the –log of the *p*-value. Dot sizes depend on the Gene Ratio as reported in the figure. **C** Proliferation rate assessed by using MTT assay in MCF-7, T-47D, ZR-75-1, MDA-MB-231 and MCF-10A cells after 72 h of lncRNA silencing. Data are presented as the mean ± SD of multiple results from a representative experiment performed in multiple replicates. Results were displayed as percentage to NC and asterisks indicate statistically significant differences using unpaired t-test (*p < 0.05, **p < 0.01, ***p < 0.005 and ****p < 0.001). **D** WB (upper panels) and relative densitometry (lower panels) showing ERα protein level following 72 h of FGD5-AS1, EPB41L4A-AS1 and PVT1 silencing or treatment with ICI (1 μM) in MCF-7, T-47D and ZR-75-1 cell lines. Tubulin (TUB) was used as loading control. Images were processed with ImageJ software (https://imagej.net) for densitometry readings. **E** Heatmap showing ERα trans-activating activity assessed in MCF-7 cells stably expressing the ERE-Luc reporter gene following lncRNAs silencing or treatment with ICI (1 μM) all in presence/absence of estrogen stimuli (10 nM). All data were analyzed with respect to the scramble NC in basal condition. Data are presented as the mean of determinations from a representative experiment performed in four independent replicates after 72 h of silencing.

A functional characterization of FGD5-AS1, EPB41L4A-AS1 and PVT1 was then performed through silencing experiments, to evaluate whether this would affect gene transcription and cell proliferation rate. Specifically, despite the three molecules were located in both cytoplasmic and nuclear compartments, isoform-specific antisense oligonucleotides (ASOs) were employed for all the experiments of this study, mainly because of their better targeting in the nucleus where the estrogen-mediated transcriptional regulation of gene expression takes place (Supplementary Fig. S3A) [38]. Transcriptome profiling following FGD5-AS1, EPB41L4A-AS1 and PVT1 knock-down identified, respectively, 1445, 818 and 1128 differentially expressed genes, compared to the effects of a scramble oligonucleotide used as negative control (Fig. 3A left, Supplementary Table S4). Among these, 304 were in common between all conditions and 86 hold at least one ERα binding site as defined by Nassa et al. [30] (Supplementary Table S5), suggesting a regulatory mechanism in which these molecules might be involved (Fig. 3A right). Furthermore, the involvement of the latter subset of deregulated genes in pathway crucial for BC progression and invasion (Fig. 3B) corroborates the possibility to consider them as putative therapeutic targets against this tumor [39, 40]. Moreover, lncRNA silencing resulted in reduction of cell proliferation rate, observed only in estrogen-responsive, ERα-positive BC cell lines MCF-7, T-47D and ZR-75-1 but not in ERα-negative MDA-MB-231 (TNBC) and MCF-10A (mammary epithelial) cells (Fig. 3C, Supplementary Fig. S3A–E). The observed functional effect was also accompanied by ERα protein reduction strongly detected particularly after silencing of PVT1 (Fig. 3D). Contrarily to what expected, based on screen score shown in Fig. 1D and on what already observed by others [41], we experienced a neglectable effect on the proliferation rate of triple negative MDA-MB-231 cells following PVT1 silencing, probably due to the isoform retrieved among ERα-enriched RNAs that was specifically targeted.

Finally, the influence of targeted lncRNA silencing on the transcriptional activity of ERα was evaluated with a trans-activation assay in hormone-deprived MCF-7 cells before and after 24 h E2 stimulation (10 nM). This resulted in a marked decrease of ERα trans-activating efficiency, once again more pronounced following PVT1 silencing (Fig. 3E), suggesting this as a possible master co-regulator of ERα-mediated transcriptional activity. On the contrary, the silencing of EPB41L4A-AS1 determined an increase of ERα activity (Fig. 3E), correlating with the demonstrated oncosuppressive role of this lncRNA [36, 41].

### The lncRNA PVT1 affects multiple BC hallmarks

**Fig. 4:**
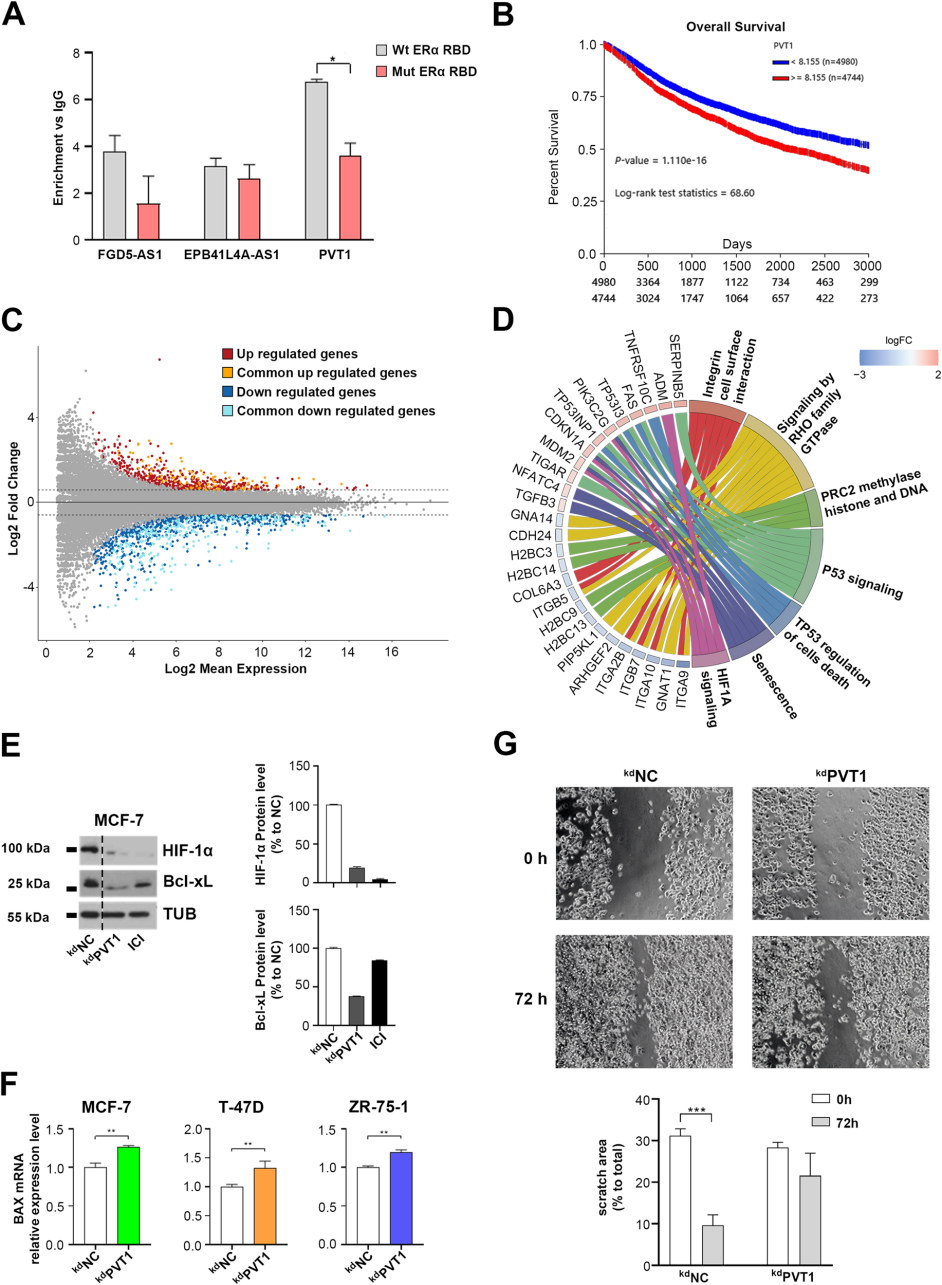
PVT1 affects multiple BC hallmarks. **A** RIP coupled to RT-qPCR showing the enrichment of ERα-associated lncRNAs in Hs-578T cell clones expressing exogenous flagged ERα wt or mutated within its predicted RNA Binding Domain (RBD). IgG was used as negative control. Data are presented as the mean of determinations from a representative experiment performed in three independent replicates after 72 h of silencing. Asterisks indicate statistically significant differences using unpaired t-test (*p < 0.05). **B** Kaplan–Meier curves, generated using UCSC Xena tool, showing the probability of overall survival in TCGA and TARGET Pan Cancer BRCA patients with low and high expression of PVT1. **C** MAPlot showing differentially expressed genes identified following PVT1 silencing in both total RNA-Seq (72 h silencing) and Nascent RNA-Seq (48 h silencing). Red and blue dots represent up and down regulated genes in RNA-Seq respectively. Orange and light blue dots represent, respectively, common up and down regulated genes in both total and Nascent RNA-Seq. **D** GO-Plot showing some of the most important pathways involving differentially expressed transcripts in total RNA-Seq data following 72 h of PVT1 silencing. Different pathways are drawn with different colors with their relative genes. LogFC ranking from -3 (blue) to 2 (red) indicates down and up regulated genes respectively. **E** WB (left panel) and relative densitometry (right panels) showing HIF-1α and Bcl-xL protein levels following 72 h of PVT1 silencing or treatment with ICI (1 μM) in MCF-7 cell line. Tubulin (TUB) was used as loading control. Densitometry results are shown as percentage to NC. Images were processed with ImageJ software (https://imagej.net) for densitometry readings. **F** RT-qPCR showing BAX mRNA relative expression level following 72 h of PVT1 silencing in MCF-7, T-47D and ZR-75-1. Results are relative to NC, used as negative control. Data are presented as the mean of determinations from three independent experiments and asterisks indicate statistically significant differences using unpaired t-test (**p < 0.01). **G** Scratch wound healing (upper) in MCF-7 at 0 and 72 h of PVT1 silencing. The percentage of scratch area was evaluated compared to total by using ImageJ software. Data are presented as the mean of determinations from three independent experiments and asterisks indicate statistically significant differences using unpaired t-test (***p < 0.005).

The comparison between nuclear ERα RIP-Seq and cytoplasmatic ERα CLIP-Seq datasets [4] allowed the identification of 17 shared lncRNAs (Supplementary Table S6), including PVT1 and FGD5-AS1, a result demonstrating their direct association with the receptor also in the cytoplasm of MCF-7 BC cell line. In order to evaluate whether this interaction would be mediated by the ERα RNA binding domain (RBD) identified by Xu et al. [4], the same constructs generated and kindly provided by these authors were used here by applying RIP coupled to RT-qPCR in a system composed of Hs-578T (ERα-negative BC cell line) stably expressing full-length-3xFlag-ESR1 (Flag-ERα) with or without mutation in its RBD. This model was chosen to avoid misleading interpretation due to hetero-dimerization between endogenous and exogenous receptor in ERα-positive cell lines. The RIP results showed a reduction of lncRNAs enrichment after nuclear flagged ERα immunoprecipitation in cells expressing mutant ERα RBD and this was significant in particular for PVT1 (Fig. 4A). We thus decided to further investigate the role of this lncRNA in regulation of estrogen-dependent gene expression, supported by the evidence that its high expression correlated with worse overall survival in BC patients (Fig. 4B). To this purpose, we went back to evaluate those genes whose expression was specifically affected by PVT1 silencing. The comparison between whole transcriptome data at 72 h of PVT1 silencing and nascent RNA profiles at 48 h after silencing identified 391 (103 up and 288 down) deregulated genes in common between the two conditions that were thus likely to be affected already in the early phases of RNA synthesis (Fig. 4C, Supplementary Table S7). These were mainly involved in pathways significant for BC progression and survival such those related to migration, response to hypoxia and cell death (Fig. 4D).

Of note, the observation of HIF-1α protein reduction corroborated the hypothesis of a deregulation of hypoxia signaling by PVT1, which has been already demonstrated to have a key role in HIF-1α stabilization in nasopharyngeal carcinoma [42]. Likewise, the reduction of the antiapoptotic protein Bcl-xL and the increase of the pro-apoptotic BAX mRNA asserted the activation of cell death response (Fig. 4E, F). In addition, a decrease in BC cell motility was also observed in the wound healing assay following PVT1 knock-down (Fig. 4G).

**Fig. 5:**
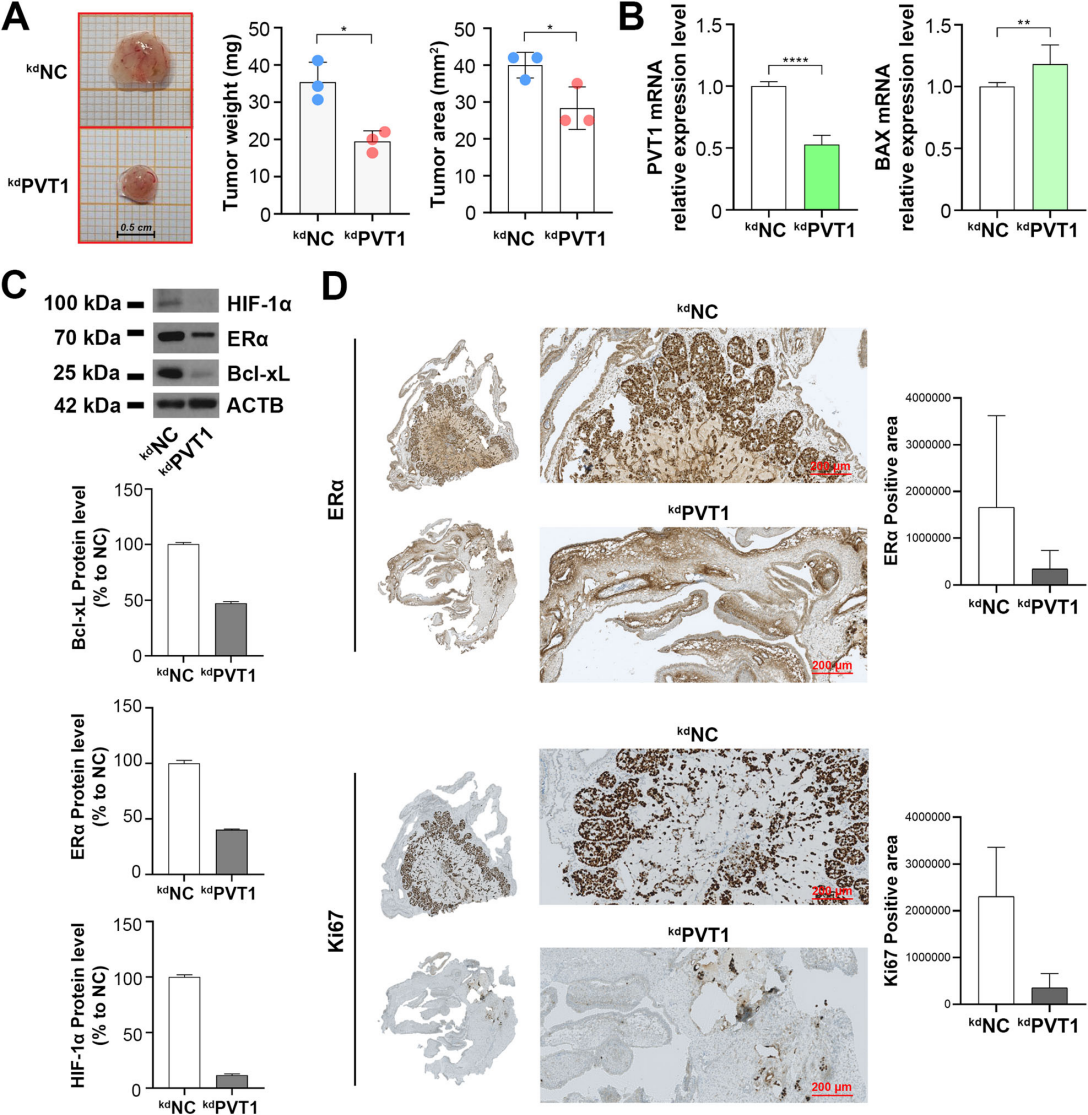
Functional characterization of PVT1 in extraembryonic CAM model. **A** Photographs of representative tumors obtained 4 days post-implantation in CAMs of MCF-7 cells silenced or not for PVT1 (left). Absolute values of weight and area (right) of tumors derived from the CAM experiment (three independent experiments were considered). Asterisk indicates statistically significant differences using unpaired t-test (*p < 0.05). **B** RT-qPCR showing PVT1 (left) and BAX (right) relative expression with or without PVT1 silencing in CAM model. NC was used as negative control. The results shown are the mean ± SD of multiple values. Asterisks indicate statistically significant differences using unpaired t-test (**p < 0.01 and ****p < 0.001). **C** WB (upper panel) and relative densitometry (lower panels) showing Bcl-xL, ERα and HIF-1α protein levels following PVT1 silencing in CAM experiments. β-actin (ACTB) was used as loading control. Densitometry results are shown as percentage to NC. Images were processed with ImageJ software (https://imagej.net) for densitometry readings. **D** Representative IHC micrographs (left) and relative quantification (right). Sections were immunostained with ERα (upper) and the proliferation marker Ki67 (lower) in different conditions. Relative quantitation was calculated on the entire section by using ImageJ software (https://imagej.net).

To further confirm the in vitro results, we employed the chicken chorioallantoic membrane (CAM) as extraembryonic in vivo model [18, 43]. On day 8th of embryo development, to achieve PVT1 silencing MCF-7 cells were in-plate transfected for 6 h, to allow the maximal effect of liposomes, as detailed in methods section, then they were detached and 5 ×10^6^ cells were inoculated into the CAMs; the tumor formation was then evaluated after 4 days. The treatment reduced both the weight and the area of the tumors (Fig. 5A). Furthermore, PVT1 silencing modulated apoptosis, as demonstrated by the increase of BAX mRNA and decrease of Bcl-xL protein levels, and hypoxia through the reduction of HIF-1α protein (Fig. 5B, C). The results were globally strengthened by a parallel reduction of ERα protein level (Fig. 5C) and confirmed after immunohistochemistry staining of FFPE samples obtained in parallel from the same tumors (Fig. 5D).

### The cooperation between PRC2 complex, PVT1 and ERα is responsible for modulation of target genes expression

**Fig. 6:**
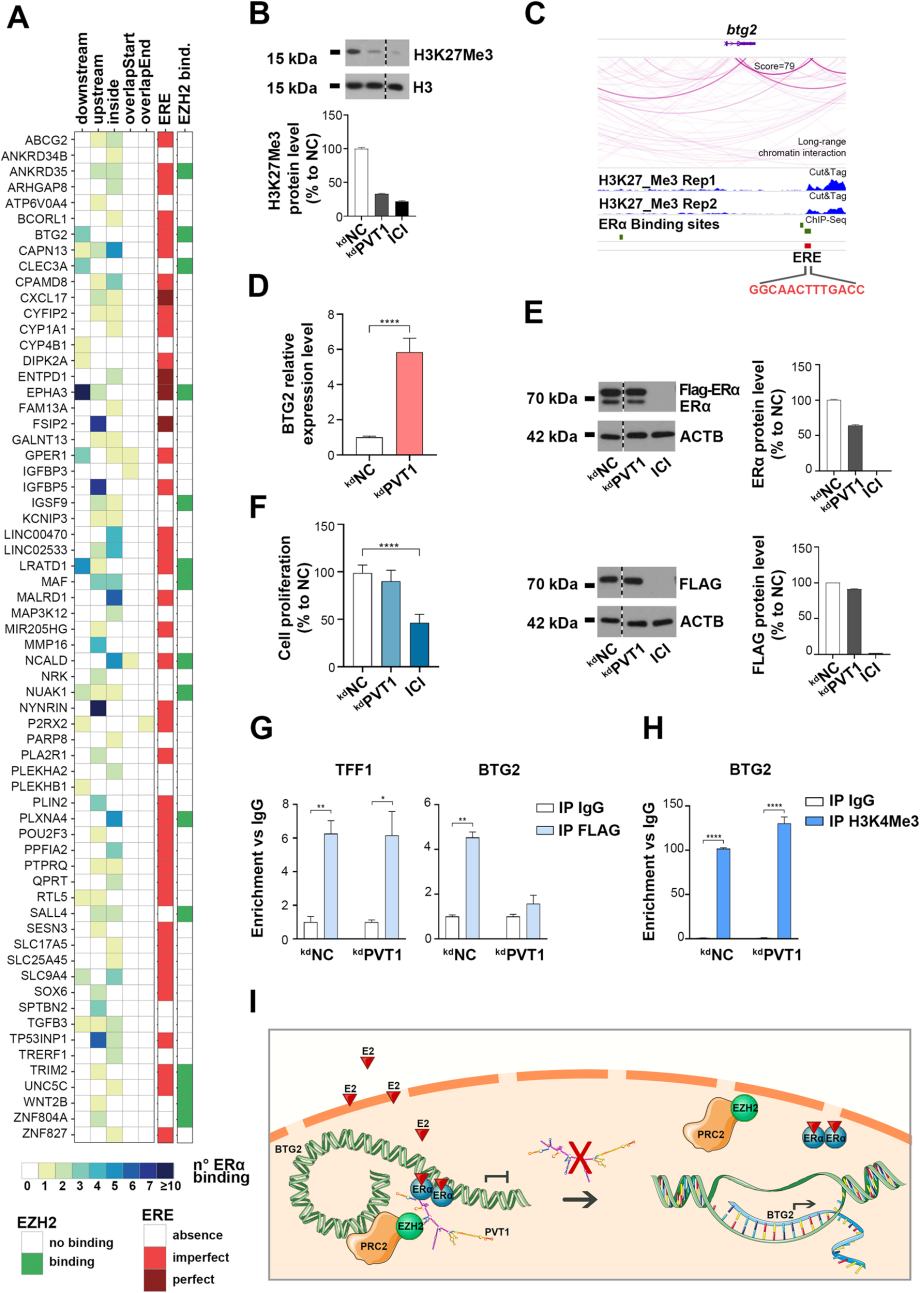
PVT1 cooperates with PRC2 and ERα for the regulation of target genes. **A** Tile plot showing 64 genes resulting downregulated following E2 stimuli and upregulated following 72 h of PVT1 silencing and holding at least one ERα binding site. Each row represents a gene; the columns indicate the genomic position of the ERα binding site followed by the presence/absence of ERE and EZH2 binding sites. The legend is reported in the lower part of the panel. **B** WB (upper panel) and relative densitometry (lower panel) showing H3K27Me3 protein level following 72 h of PVT1 silencing or treatment with ICI (1 μM) in MCF-7 cells. H3 was used as control. Densitometry results are shown as percentage to NC. Images were processed with ImageJ software (https://imagej.net) for densitometry readings. **C** Integrative Genomics Viewer (IGV) view of the long-range chromatin interactions of CHIA-PET experiments, the two replicates of H3K27Me3 Cut&Tag experiment showing BTG2 surroundings, the imperfect ERE located on it and the nearest ERα binding site. **D** RT-qPCR showing BTG2 relative expression with or without PVT1 silencing in CAM model. NC was used as negative control. The results shown are the mean ± SD of multiple values. Asterisks indicate statistically significant differences using unpaired t-test (****p < 0.001). **E** WBs (left panels) and relative densitometry (right panels) showing ERα and FLAG protein level following 72 h of PVT1 silencing or treatment with ICI (1 μM) in MCF-7 clones. β-actin (ACTB) was used as control. Densitometry results are shown as percentage to NC. Images were processed with ImageJ software (https://imagej.net) for densitometry readings. **F** Proliferation rate assessed by using MTT assay in MCF-7 clones stably expressing exogenous Flag-ERα after 72 h of silencing. Data are presented as the mean ± SD of multiple results from a representative experiment performed in multiple replicates. Results were displayed as percentage to NC and asterisks indicate statistically significant differences using unpaired t-test (****p < 0.001). **G** ChIP coupled to qPCR displaying the recruitment of Flag-ERα on TFF1 and BTG2 genes with or without 72 h of PVT1 silencing. IgG was used as negative control. The results showed are the mean ± SD of triplicate values. Asterisks indicate statistically significant differences using unpaired t-test (*p < 0.05 and **p < 0.01). **H** ChIP coupled to qPCR displaying the enrichment of H3K4Me3 on BTG2 gene in presence or absence of 72 h of PVT1 silencing. IgG was used as negative control. The results showed are the mean ± SD of triplicate values. Asterisks indicate statistically significant differences using unpaired t-test (****p < 0.001). **I** Schematic representation of the proposed model involving PRC2, ERα and PVT1 on BTG2 gene expression regulation.

Transcriptome analysis following PVT1 silencing displayed, among deregulated ones, the pathway related to PRC2 activity (Fig. 4D). Since it was previously described the association between PVT1 and the PRC2-subunit EZH2 [44, 45] and given that the interaction between EZH2 and ERα was demonstrated to be mediated by RNAs (Fig. 1A), we hypothesized a nuclear cooperating machinery involving ERα, EZH2 and the lncRNA PVT1. Based on the knowledge that EZH2-mediated trimethylation of H3K27 is a marker of gene silencing, we speculated a repression machinery involving these three factors. For this reason, we searched among genes downregulated following estrogen stimulation and those upregulated following PVT1 silencing, identifying 124 common targets involved in BC-related hallmarks such as hypoxia and oxidative stress response, p53 and K-Ras pathway (Supplementary Fig. S4A-B, Supplementary Table S8). Among them, 64 displayed at least one ERα binding site in their transcription unit, 41 of which holding a perfect/imperfect ERE and 15 an EZH2 binding site (Fig. 6A). We then investigated whether PVT1 could affect the level of the main protein components of the PRC2 complex (JARID2, EZH2, SUZ12 and PHF1), but no differences were observed before and after its silencing (Supplementary Fig. S4C). Interestingly, a strong reduction in H3K27Me3, the histone modification specifically induced by EZH2 was observed following PVT1 knock-down (Fig. 6B), indicating that indeed, as predicted by functional analysis, this lncRNA is likely to modulate PRC2 activity by affecting this histone mark.

Among the genes commonly occupied by ERα and EZH2, the tumor suppressor BTG2 was selected for validation, based on its crucial role as cancer antagonist [46, 47]. Analysis of publicly available data from CUT&Tag experiments [28], performed in cell models and experimental conditions comparable to ours, showed an abundance of H3K27Me3 markers both upstream and downstream BTG2 gene, which is more pronounced in correspondence of the ERα-bound imperfect ERE motif (Fig. 6C). Furthermore, CHIA-PET experiments (GSE176821) focusing on the long-range chromatin interactions show a good interaction score (79) between BTG2 and the ERE motif in the investigated genomic region, despite the presence of another ERα binding site in BTG2 upstream region. The hypothesized hormone depending trans-repression complex induces a downregulation of BTG2 mRNA following estrogen stimulation (Supplementary Fig. S4D), while the blockade of either EZH2 or ERα, using GSK126 and Tamoxifen respectively, causes an overexpression of the transcript (Supplementary Fig. S4E), confirming that both these factors are needed for BTG2 trans-repression. Moreover, also PVT1 silencing induces an upregulation of BTG2 transcript in cell lines and in the CAM model (Fig. 6D, Supplementary Fig. S4F). In this view, we aimed to investigate whether the observed modulation was mediated by the lncRNA PVT1. To avoid confounding results depending upon PVT1-induced endogenous ERα modulation, we employed a MCF-7 cell clone expressing an exogenous Flag-ERα whose expression was mostly unaffected by PVT1 silencing, as also demonstrated by the neglectable effect observed on cell proliferation (Fig. 6E, F, Supplementary Fig. S4G). Using this approach, the immunoprecipitation of Flag-ERα following PVT1 silencing indicated a reduced recruitment of the receptor on BTG2 gene compared to TFF1 promoter, used as control (Fig. 6G). In addition, the trimethylation of H3K4, that is a marker of transcriptionally active chromatin, was significantly more pronounced on BTG2 following PVT1 silencing (Fig. 6H). Based on the results described here, we propose a mechanistic model for E2 depending BTG2 transcriptional repression in which ERα needs the recruitment of PRC2 for EZH2-mediated H3K27Me3 and chromatin closure and PVT1 acts as a bridging factor between the two functional components (Fig. 6I).

## Discussion

ERα is a mitogenic transcription factor in BC onset and progression by modulating the expression of responsive target genes in luminal-like BC cells [48].

Wide dissection of chromatin-associated ERα multiprotein complexes has been performed in several experimental conditions leading to partial definition of the nuclear dynamics associated with standard therapies response or resistance in hormone-dependent BCs [49, 50]. Nevertheless, the discovery of the receptor’s ability to act as an RNA-binding protein [4] has opened new perspectives in the possibility to identify new molecules bridging protein-protein interactions in transcriptional modulation of gene expression that would guide alternative multi-molecule complexes formation in transcriptional activation and silencing.

Starting from these evidences and from our previous studies, demonstrating the existence of a RNA-mediated ERα interactome [31], we performed native nuclear ERα-associated RNA immunoprecipitation and sequencing to identify novel molecules involved in transduction of the estrogenic signaling to chromatin.

Among ERα interacting RNAs identified, we focused on lncRNAs because of their known roles in transcriptional modulation of gene expression. Indeed, although the known functional relationship between ERα and several lncRNAs in BC [7], there was no clear evidence demonstrating physical association of the receptor with these regulatory molecules so far. Comparing the dataset generated here with previously published data identifying functional lncRNAs in multiple cancer models [17], FGD5-AS1, EPB41L4A-AS1 and PVT1 were selected for further experimental evaluations based on their fitness score demonstrated in BC cell models, average expression and ERα-enrichment in our experimental models (Fig. 1D, E).

After a first functional evaluation, indicating specific expression and intracellular compartmentalization for each molecule, we observed that all of them were able to associate with BC cell chromatin and participate in transcriptional transduction of the estrogenic signaling, since their silencing determined deregulation of estrogen-responsive genes and inhibition of ERα-positive BC cell proliferation (Fig. 3A–C). The stronger effect observed for PVT1, its positive correlation with ERα protein expression and trans-activating function and the significant inverse correlation with BC patients’ overall survival, suggested this as a nodal factor from the mechanistic point of view (Figs. 3D, E and 4B). Indeed, although PVT1 has been functionally studied and demonstrated to act as a pan-oncogene in several cancer models, including BC, its direct and nuclear functional association with ERα and the estrogenic signaling has not been investigated so far. By comparing the functional effects elicited by PVT1 silencing in our models with what already known, we hypothesized that this lncRNA could act as a core molecule in the functional cooperation between ERα-mediated transcriptional regulation and the activity of the PRC2 complex in suppression of specific genes. Indeed, it has been recently demonstrated that PVT1 acts as a molecular partner of PRC2 in multiple myeloma through its physical interaction with the enzymatic subunit EZH2, that catalyzes H3K27 trimethylation [38], and it was previously shown that PVT1 is involved in non-small cell lung cancer metastasis through EZH2 [51].

On the other hand, EZH2 expression has been demonstrated to be modulated by ERα in BC [52], where both are involved within a transcriptional axis with GREB1 in induction of tamoxifen resistance [53]. Moreover, EZH2 was among ERα partners whose association with the receptor was found to be RNA-dependent [31] (Fig. 1A).

To strengthen previous observations, “PRC2 methylase histone and DNA” was among the functional pathways significantly affected when considering ERα target gene deregulation induced by PVT1 silencing (Fig. 4D). To investigate the possible existence of a multi-modular transcriptional repression complex involving ERα, PRC2 and PVT1, we focused on ERα down-regulated genes whose expression was affected by PVT1 kd and that were characterized from the presence of both ERα and EZH2 binding sites within their transcription units (Fig. 6A). Among them, BTG2 caught our attention, as it has been demonstrated to be an estrogen/ERα down-regulated tumor suppressor significantly associated with low survival rate in luminal-like BC, and proposed as possible molecular target for the treatment of these tumors [46, 54]. We speculated that, as previously observed in different cancer models, PVT1 binds to and carries PRC2 complex to target genomic regions to suppress transcription of specific genes. In hormone-responsive BC cells, this would be achieved through the coordinated recruitment of EZH2 involving ERα-associated multi-molecules complexes to down-regulate pro-apoptotic and/or tumor suppressor genes such as BTG2, thus allowing cell proliferation and invasion reduction. Given that, in the proposed model, EZH2 and ERα proteins are both required for target genes modulation, disrupting their association through PVT1 inhibition might be a useful tool for blocking anti-estrogen resistant cell proliferation, since both PVT1 and EZH2 have been already involved in resistance to therapy in several models [55–57]. The recognized therapeutic value of ASO administration will now make it possible to consider PVT1 silencing as a novel pharmacological tool to be used alone or in combination with standard therapies in the treatment of ERα-positive BCs.

## Supplementary information

Supplemental methods and figures

Supplemental table 1

Supplemental table 2

Supplemental table 3

Supplemental table 4

Supplemental table 5

Supplemental table 6

Supplemental table 7

Supplemental table 8

Original wester blot images

## Data Availability

The datasets generated and/or analyzed in the current study are available in the ArrayExpress repository with the following accession numbers: E-MTAB-14147, E-MTAB-14148, and E-MTAB-14149. Uncropped original western blots are available as Supplementary Material.
