# Supplementary material for: LncRNA PVT1 links estrogen receptor alpha and the polycomb repressive complex 2 in suppression of pro-apoptotic genes in hormone-responsive breast cancer
Source: Cell Death Dis. 2025 Feb 8;16(1):80. doi: 10.1038/s41419-025-07423-4 (PMC11807188; doi:10.1038/s41419-025-07423-4)
Supplement: Supplementary file 1 — Supplemental methods and figures [file 41419_2025_7423_MOESM1_ESM.docx]

**1. Supplementary Methods**

**1.1 Cell Culture**

The human BC cell lines MCF-7 (HTB-22), T-47D (HTB-133), ZR-75-1(CRL-1500), MDA-MD-231 (HTB-26), Hs-578T (HTB-126) and the epithelial breast cell line MCF-10A (CRL-10317) were purchased from the American Type Culture Collection (ATCC) and cultured according to manufacturer’s protocol. MCF-7 stably expressing ERE-TK-LUC (luciferase reporter gene under the control of estrogen responsive element (ERE)) [(1)](https://sciwheel.com/work/citation?ids=1237678&pre=&suf=&sa=0&dbf=0) used for transactivation assay, were cultured in Dulbecco's modified Eagle’s medium (Euroclone, Milan, Italy) supplemented with 10% FBS (HyClone, Milan, Italy) and 100 U/ml penicillin (Euroclone), 100 mg/ml streptomycin (Euroclone), 2.5 mM L-glutamine (Euroclone) and 250 ng/ml Amphotericin-B (Sigma-Aldrich, Milan, Italy). Estrogen deprivation was performed by culturing cells for 5 days in phenol-red free medium supplemented with 5% dextran-coated charcoal treated serum. Hs-578T-FlagERα clones were generated by stably transfecting a full-length-3xFlag-ESR1 plasmid with *wt* or mutant ERα RNA binding domain (RBD); the constructs were kindly provided by Dr. Ruggero [(2)](https://sciwheel.com/work/citation?ids=11741011&pre=&suf=&sa=0&dbf=0). All cell lines were authenticated by STR profiling and routinely tested for mycoplasma contamination.

**1.2 Antibodies and compounds**

The antibodies employed for immunoprecipitation experiments were: anti-ERα (ab3575, Abcam, Cambridge, UK), Rabbit IgG Isotype Control (31235, Thermo Fisher, Milan, Italy), anti-FLAG M2 (F1804, Sigma Aldrich), anti-Mouse IgG (ab190475, Abcam) and anti-Histone H3 trimethyl K4 (ab8580, Abcam).

The antibodies used for western blot experiments were: anti-ERα (F-10 sc-8002, Santa Cruz Biotechnology, Dallas, Texas, USA), anti-menin (A300–105 A, Bethyl Laboratories, Montgomery, Texas, USA), anti-WSTF (BAZ1B; A300-446A, Bethyl Laboratories), anti-β-actin (A1978, Sigma-Aldrich), anti-HIF-1α (A22041, ABclonal, Massachusetts, USA), anti-FLAG M2 (F1804, Sigma Aldrich), anti-α-Tubulin (sc-32293, Santa Cruz Biotechnology), anti-Bcl-xL (#2764, Cell Signaling, Danvers, Massachusetts, USA), anti-Histone H3 (ab1791, Abcam), anti-Histone H3 trimethyl K27 (ab6002, Abcam), anti-SUZ12 (ab12073, Abcam), anti-JARID2 (D6M9X, Cell Signaling), anti-PHF1 (ab184951, Abcam) and anti-EZH2 (#5246, Cell Signaling).

The antibodies utilised for immunohistochemistry were: anti-HIF-1α (A22041, ABclonal), anti-ERα (ab3575, Abcam) and anti-Ki67 (ab16667, Abcam).

Compounds used for cell treatments were 17β-estradiol (Cat. E887-5G, Sigma-Aldrich), 4-hydroxytamoxifen (TAM; Cat. H7904, Sigma-Aldrich), fulvestrant (ICI; Cat. I4409, Sigma-Aldrich) and GSK126 (CAY-15415-5, Chemgood, Henrico, Virginia, USA).

**1.3 Protein extraction, Immunoprecipitation and Western Blot assay**

Total proteins and subcellular protein extraction were performed as previously described [(3)](https://sciwheel.com/work/citation?ids=7418011&pre=&suf=&sa=0&dbf=0)[(4)](https://sciwheel.com/work/citation?ids=14411898&pre=&suf=&sa=0&dbf=0). Protein concentrations were determined using Bradford assay and their expression was analysed by western blotting, according to standard protocols. Densitometry was performed by ImageJ software analysis [(5)](https://sciwheel.com/work/citation?ids=222322&pre=&suf=&sa=0&dbf=0).

For immunoprecipitation 2.5 µg of anti-ERα and Rabbit IgG Isotype Control (Thermo Fisher) were conjugated with 35 µl of equilibrated Dynabeads M-280 Sheep AntiRabbit IgG (Thermo Fisher), in rotation overnight at 4°C and the experiment was performed as previously described [(6)](https://sciwheel.com/work/citation?ids=13397899&pre=&suf=&sa=0&dbf=0). Nuclear protein extracts were treated with 100 μg/ml RNaseA (Cat. 12091021, Invitrogen, Massachusetts, USA) and incubated 1 h at 4 °C with gentle rotation before binding. Immunoprecipitation was performed incubating conjugated beads/antibodies at 4°C for 2 h with 500 µg of nuclear protein extracts. After the incubation, beads were washed with IPP150 buffer (7.14 mM HEPES pH 7.5, 8.92% glycerol, 150 mM NaCl, 0.54 mM MgCl2, 0.07 mM EDTA pH 8, 1x PIC), wash buffer (50 mM Tris-HCl pH 7.6, 150 mM NaCl and 1x PIC) and resuspended in Laemmli buffer for western blot assay.

**1.4 RT-qPCR**

RNA was reverse transcribed to complementary DNA (cDNA) using cDNA Synthesis Kit (BIO-65054, Meridian Bioscience, Cincinnati, Ohio, USA) according to manufacturer’s instructions. RT-qPCRs were performed, in triplicate, using SensiFAST SYBR Lo-ROX kit (BIO-94020, Meridian Bioscience) on QuantStudio 7 Pro (Applied Biosistems, Thermo Fisher). Relative RNAs expression was calculated by standard 2^-ΔΔCt^ method. The housekeeping gene RPLP0 was used as internal control for total RNA samples while MALAT1 and GAPDH were used as controls for nuclear and cytoplasmic RNA fraction respectively. The sequences of primers are listed below:

| ESR1 | Forward Primer: ACCCTCCATGATCAGGTCCA  Reverse Primer: CTGGTTCCTGTCCAAGAGCA |
| --- | --- |
| FGD5-AS1_1 | Forward Primer: TGAAGGGCTGGTCGTCCTAA  Reverse Primer: CAACAGACACTTTGCGAGCC |
| FGD5-AS1_2 | Forward Primer: TCTGGCATCAGCACTTTCACT  Reverse Primer: GCAACGACCTGTCTCTCTGAA |
| EPB41L4A-AS1_1 | Forward Primer: CGACTATGCCAGGGAGTTCT  Reverse Primer: GGGCAGGGCAAGCATAAAGT |
| EPB41L4A-AS1_2 | Forward Primer: CTGACTTTATGCTTGCCCTGC  Reverse Primer: GATCACTCCCGACGAGCAC |
| PVT1_1 | Forward Primer: TGTGGCTGAATGCCTCATGG  Reverse Primer: GACCCAAAGGAAGTTGGCAG |
| PVT1_2 | Forward Primer: CATGCACTGGAATGACACACG  Reverse Primer: ATCTCAACCCTCTCAGCCAG |
| MALAT1 | Forward Primer: TGGTGATGAAGGTAGCAGGC  Reverse Primer: CATATTGCCGACCTCACGGA |
| GAPDH | Forward Primer: CACATGGCCTCCAAGGAGTAA  Reverse Primer: TGAGGGTCTCTCTCTTCCTCTTGT |
| BAX | Forward Primer: TGGCAGCTGAACATGTTTTCTGAC  Reverse Primer: TCACCCAACCACCCTGGTCTT |
| RPLP0 | Forward Primer: CCATCAGCACCACAGCTTC  Reverse Primer: GGCGACCTGGAAGTCCAACT |
| PNRCR1 | Forward Primer: TCTCACCCTTCTTTTCAGGATTG  Reverse Primer: TTGTCTTGGCAGAGGACTTTCT |
| TFF1 | Forward Primer: GTGGTTTTCCTGGTGTCACG  Reverse Primer: TCACACTCCTCTTCTGGAGGG |
| TFF1_promoter | Forward Primer: AGCAGGAAGAAGCACGCCTTA  Reverse Primer: GACTCGGGGTCGCCTTTG |
| BTG2 | Forward Primer: CTAGGCATCTGACCTCGGTG  Reverse Primer: TGGGCTGAGTGGAGGTAAGT |

**1.5 RNA extraction**

Nascent RNAs [(7)](https://sciwheel.com/work/citation?ids=1558660&pre=&suf=&sa=0&dbf=0) and fractionated RNAs from cytosol and nucleus [(8)](https://sciwheel.com/work/citation?ids=7944433&pre=&suf=&sa=0&dbf=0) were processed from each sample as previously described. RNAs were extracted using TRIzol^TM^ (Life Technologies, Thermo Fisher), according to the manufacturer’s instructions.

**1.6 CARIP assay**

Chromatin associated RNAs immunoprecipitation (CARIP) was performed in accordance with Kidder’s protocol [(9)](https://sciwheel.com/work/citation?ids=6862370&pre=&suf=&sa=0&dbf=0) with minor modifications. 10 μg of anti-ERα or anti-IgG Isotype Control antibodies were conjugated with 50 μl of Dynabeads M-280 Sheep AntiRabbit IgG (Thermo Fisher) in rotation overnight at 4°C. Cross-linking was performed on MCF-7 cells by adding 1% formaldehyde for 10 min at room temperature. Then, the quenching was performed by adding 125 mM glycine for 8 min at room temperature. Cells were washed, harvested by scraping in cold PBS and overnight stored at -80 °C. Nuclear isolation was performed by resuspending cell pellet in Lysis Buffer I (50 mM Hepes KOH pH 7.5, 140 mM NaCl, 1 mM EDTA, 10% glycerol, 0.5% NP-40, 0.25% Triton-X-100, 1x PIC, 100 U/ml RNAse inhibitor) and incubating the sample for 10 min with gentle rotation at 4 °C. After a centrifugation at 2000 x g for 3 min at 4°C the resulting supernatant was discarded and the nuclear pellet was resuspended in Lysis Buffer II (10 mM Tris HCl pH 8.0 200 mM NaCl, 1 mM EDTA, 0.5 M EGTA, 1x PIC, 100 U/ml RNAse inhibitor), incubated with gentle rotation for 5 min and centrifuged at 2000 x g for 3 min at 4°C. The cell pellet was resuspended in Lysis Buffer III (10 mM Tris HCl pH 8, 100 mM NaCl, 1 mM EDTA, 0.1% Na-Deoxycholate, 0.5% N-lauroylsarcosine, 1 x PIC, 100 U/ml RNAse inhibitor) and sonicated for 10 cycles (30’’ ON and 30’’ OFF) using Bioruptor (Diagenode). Subsequently, Lysis Buffer III containing 10% of Triton-X-100 was added and the sample was centrifuged at full speed for 10 min at 4°C. The resulting supernatant represents the nuclear crosslinked fraction, and an aliquot of this extract was taken as input for DNA, RNA and protein analysis before binding. CARIP immunoprecipitation, de-crosslinking and DNA extraction were performed in accordance with Kidder’s protocol[(9)](https://sciwheel.com/work/citation?ids=6862370&pre=&suf=&sa=0&dbf=0) while RNA extraction was achieved using TRIzol^TM^ (Life Technologies, Thermo Fisher) according to the manufacturer’s guideline.

**1.7 Knock-down experiments**

Cell lines were seeded into 96-well (15000 cells/well), 12-well (200000 cells/well) or 6-well (500000 cells/well) plates in standard growth medium and, the day after, transiently transfected by using Lipofectamine RNAiMax (Cat. 13778–150, Invitrogen) in OptiMem medium (Gibco) according to the manufacturer’s instructions. For siRNA-mediated knock-down, 0.5pM/well of ESR1 (s4825) or scramble Silencer Select Negative Control (SiSel_NC1) (Ambion, Thermo Fisher Scientific) were added to the cells in quadrupled/sixfold each one and incubated for 72 h at 37 °C/5% CO_2_. ASO-mediated knock-down was employed for lncRNAs silencing (PVT1 #231193149, FGD5-AS1 #231193147, EPB41L4A-AS1 #231193150 and NC #231193151, IDT): 3, 30 or 75 pMol/well of ASOs were transfected for 96-well, 12-well and 6-well plates respectively and incubated for 48 or 72 h at 37 °C/5% CO_2_.

**1.8 Cell proliferation assays**

MTT assay was applied for cell proliferation rate assessment. Briefly, cells were sixfold seeded at a density of 15000 cells/well into 96-well plates. Following 72 h of silencing, cell proliferation was evaluated by adding 1 mg/ml of 3-(4,5-dimethylthiazol-2-yl)-2,5-diphenyltetrazolium bromide (MTT) (Cat. M6494, Thermo Fisher) and incubated for 4 h at 37 °C/5% CO_2_. The formazan crystals were solubilised by adding 100 μl/well of 0.4 M HCl in 2-propanol and the absorbance was measured at 570 nm and 620 nm for test and background respectively by using Spark multimode microplate reader (TECAN, Männedorf, Switzerland).

**1.9 Luciferase (Trans-activation) assay**

Cells were plated in quadruplicates in 96-well plates at a density of 15000 cells/well and silenced as described before. After 72 h, cells were harvested in lysis buffer (Cat. E153A, Promega, Madison, Wisconsin, USA) and luciferase activity was quantified using the Luciferase Assay Reagent (Cat. E1500, Promega) according to the manufacturer’s instructions. The values achieved were subsequently normalized to the protein concentrations measured using the Bradford assay.

**1.10 Wound-healing assay**

200000 cells/well were seeded in 12-well plates and cultured until their confluency reached 90-100%. A 200-μl sterile pipette tip was used to create a scratch across the middle of each well. Cells were silenced as described before. The inversion microscope Leica DMI4000B and the Leica Application Suite v 4.7 were used to capture images at 0 and 72 h of silencing. Scratch areas were calculated employing ImageJ software analysis [(5)](https://sciwheel.com/work/citation?ids=222322&pre=&suf=&sa=0&dbf=0).

**1.11 Immunohistochemistry (automated protein detection)**

IHC standardized assays were performed in 5-µm paraffin sections with VENTANA BenchMark Ultra automated staining instrument (Ventana Medical Systems, Roche, Basel, Switzerland), using VENTANA reagents except as noted, according to the manufacturer's instructions. Slides were deparaffinized using EZ Prep solution (cat # 950–102) for 16 min at 72 °C. Epitope retrieval was accomplished with CC1 solution (cat # 950–224) at a high temperature (95°C) for a period that is suitable for a specific tissue type. Antibodies were titered with a blocking solution into user fillable dispensers for use on the automated stainer. Slides were developed using the VENTANA ultraView Universal DAB detection kit (cat #760-500) according to the manufacturer’s instructions. Slides were then counterstained with hematoxylin II (cat # 790-2208) for 8 min, followed by Bluing reagent (cat # 760-2037) for 4 min. Bright-field section were scanned with ZEISS Axio Scan (ZEISS, Oberkochen, Germany).

**1.12 Histone Extraction**

Cells were harvested, washed twice in cold PBS and resuspended in Triton Extraction Buffer (TEB: PBS containing 0.5% Triton X-100 (v/v), 2 mm PMSF, 0.02% (w/v) NaN3) and lysed at 4°C for 10 min with gentle stirring. The nuclear fraction was achieved by centrifugation at 2000 rpm for 10 min at 4°C. Nuclei were then suspended in 0.2 N HCl and incubated overnight at 4°C on a stirrer. Subsequently, samples were centrifuged and the supernatant, containing histones, was quantified using Bradford assay [(10)](https://sciwheel.com/work/citation?ids=12631494&pre=&suf=&sa=0&dbf=0).

**1.13 Chromatin immunoprecipitation**

For Chromatin immunoprecipitation (ChIP), 50 μl of Dynabeads M-280 Sheep Anti-Rabbit IgG (Thermo-Fisher) or Dynabeads M-280 Sheep Anti-Mouse IgG were overnight incubated at 4°C with 5 μg of the specific antibodies and the protocol was perfomed as previously described [(6)](https://sciwheel.com/work/citation?ids=13397899&pre=&suf=&sa=0&dbf=0).

DNA was eluted twice by adding directly on the beads 0.2 ml of Elution buffer (50 mM TrisHCl pH 8, 10 mM EDTA, 1% SDS) for 30 min at 25°C with gentle shaking. Decrosslinking was performed by adding 16 μl of NaCl 5M and overnight incubating the sample at 65°C. Eluted DNA was treated with RNase A (20 ng/ml) for 15 min at 37°C followed by proteinase K (200 ng/ml) for 4 h 55°C. Finally, DNAs were purified by phenol-chloroform extraction.

**2. Supplementary Figures**


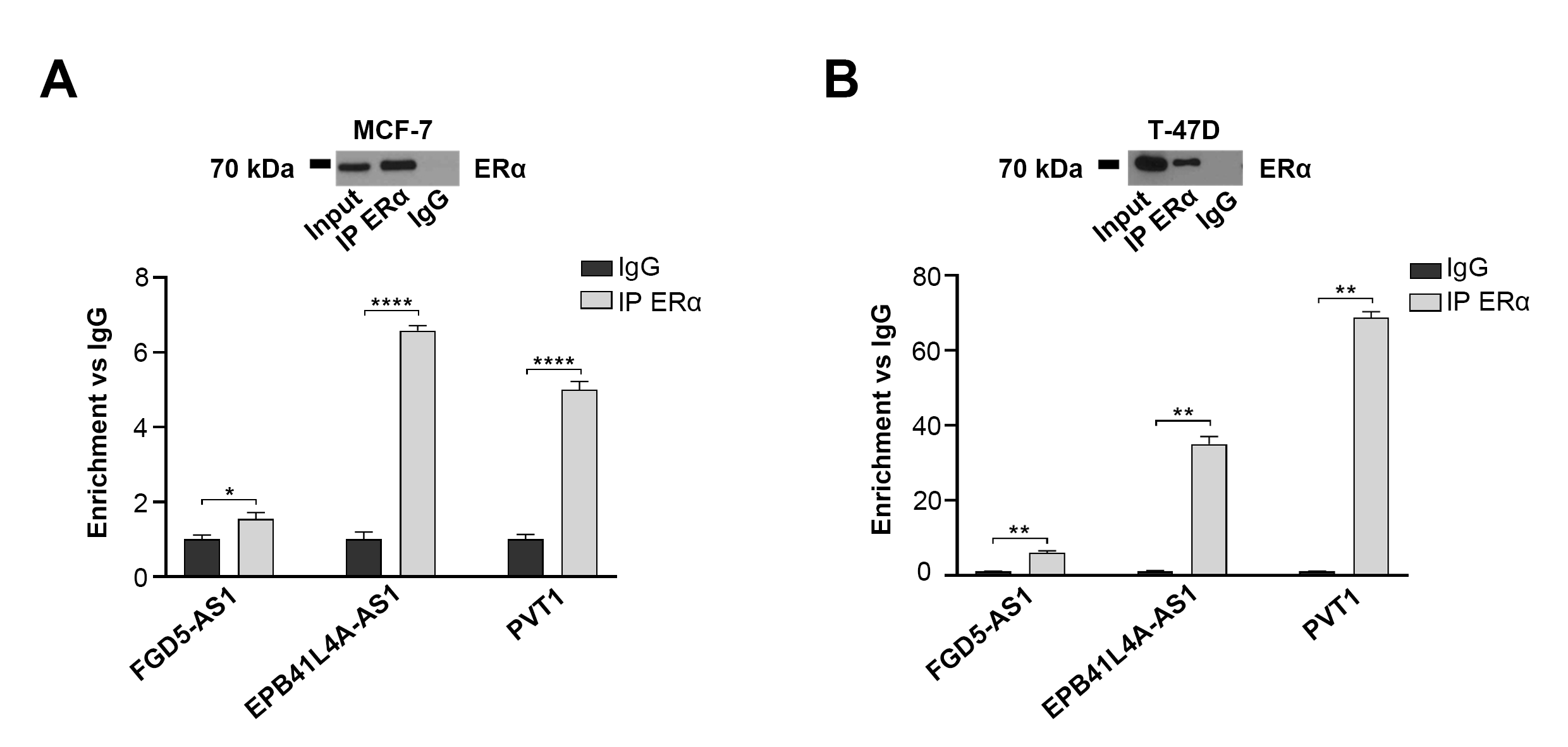


**Figure S1 Validation of ERα**-**enriched** **lncRNAs in alternative cell models.** RIP coupled to RT-qPCR validating the enrichment of the three ERα-associated lncRNAs in MCF-7 **A)** and T-47D **B)** BC cell nuclei using IgG as negative control. The upper panels of the figure show WB confirming ERα immunoprecipitation along RIP. The results showed are the mean ± SD of triplicate values. Asterisks indicate statistically significant differences using unpaired t-test (* p <0.05, ** p <0.01 and **** p <0.001).


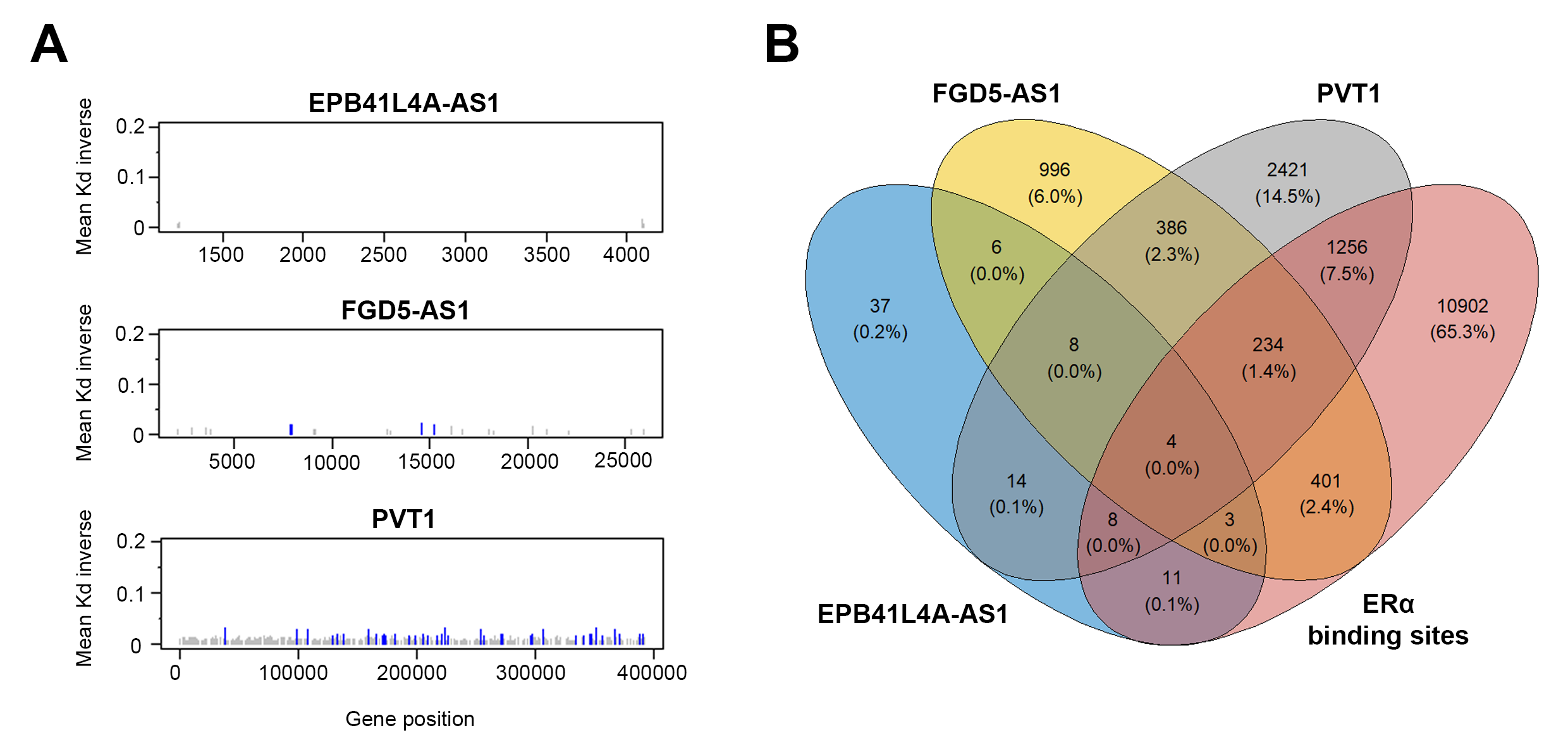


**Figure S2 Genomic and epigenetic characterization of FGD5-AS1, EPB41L4A-AS1 and PVT1 A)** Plot showing EREFinder results. On the x-axis is shown the position on the gene surroundings and on the y-axis the Mean Kd inverse value. Blue bars represent significant peaks. **B)** Venn diagram showing the intersection between Long-Target results, predicting genomic binding sites of the three lncRNAs, and the ERα binding sites.


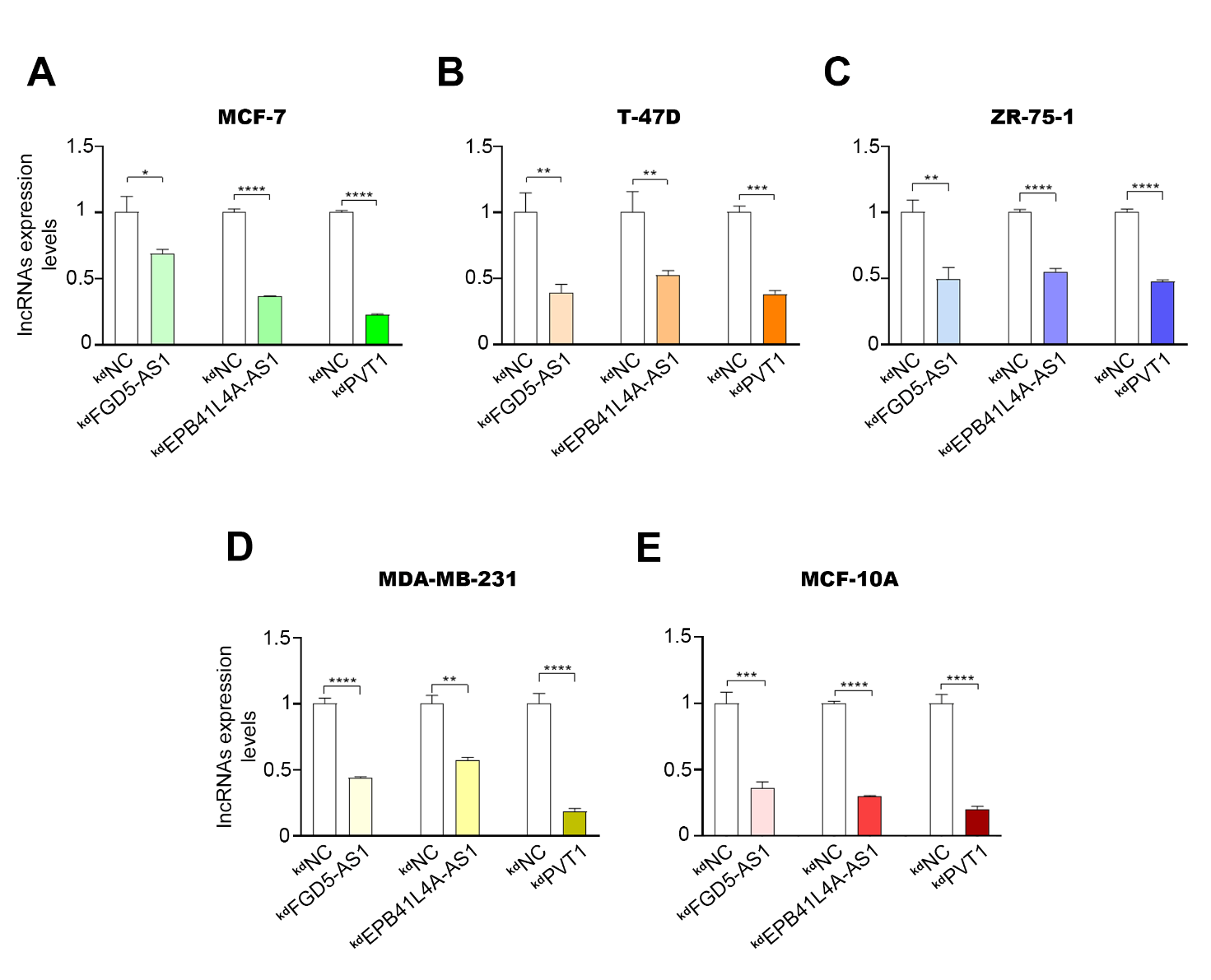


**Figure S3 lncRNAs silencing efficiency** RT-qPCR showing the silencing efficiency of FGD5-AS1, EPB41L4A-AS1 and PVT1 after 72 h of ASO transfection in MCF-7 **(A)**, T-47D **(B)**, ZR-75-1 **(C)**, MDA-MB-231 **(D)** and MCF-10A **(E)** cells. Results are relative to NC, used as negative control. Data are presented as the mean of determinations from a three independent experiment and asterisks indicate statistically significant differences using unpaired t-test (* p <0.05, ** p <0.01, *** p <0.005 and **** p <0.001).


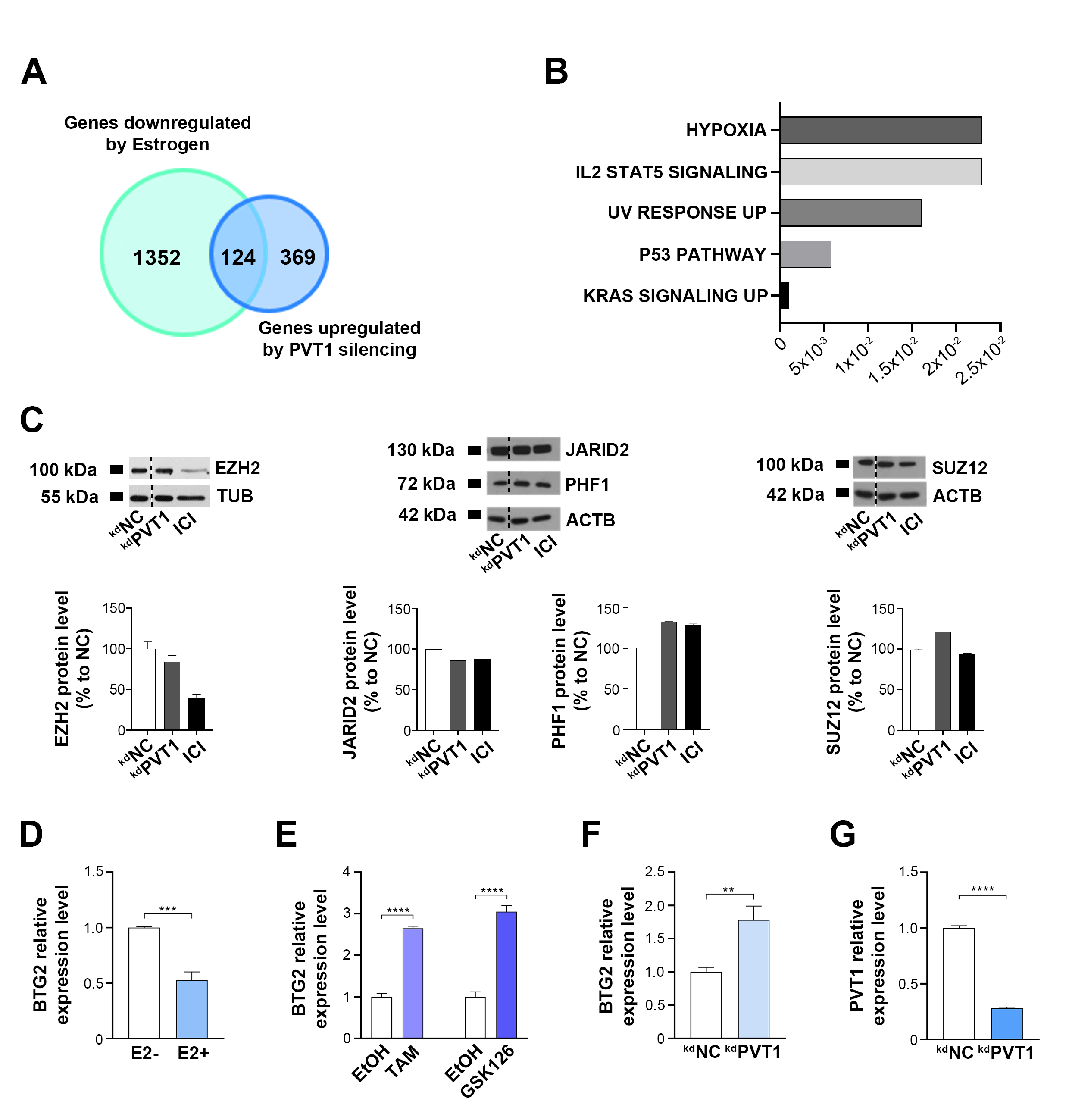


**Figure S4** **PVT1 regulates tumor suppressor genes** **through ERα and PRC2 complex** **A)** Venn diagram showing the intersection between genes downregulated by estrogen and those upregulated by PVT1 silencing and **B)** Gene Set Enrichment Analysis (GSEA) showing the principal hallmarks in which those are involved. **C)** WBs (upper panels) and relative densitometry (lower panels) showing EZH2, JARID2, PHF1 and SUZ12 protein level following 72 h of PVT1 silencing or treatment with ICI (1 μM) in MCF-7 cells. β-actin (ACTB) or tubulin (TUB) were used as controls. Densitometry results are shown as percentage to NC. Images were processed with ImageJ software (https://imagej.net) for densitometry readings. RT-qPCR showing BTG2 expression levels with or without estrogen stimulation **(D)**, with 72 h of Tamoxifen (1 μM) or GSK126 (1 μM) treatment **(E)** and following 72 h of PVT1 silencing **(F)**. Ethanol (EtOH) or scramble were used as negative controls. The results showed are the mean ± SD of triplicate values. Asterisks indicate statistically significant differences using unpaired t-test (** p < 0.01, *** p <0.005, **** p <0.001). **(G)** RT-qPCR showing PVT1 72 h silencing in MCF-7 clone. Results are relative to NC, used as negative control. Data are presented as the mean of determinations from three independent experiments and asterisks indicate statistically significant differences using unpaired t-test (**** p <0.001).

**References**

[1.    Balaguer P, Boussioux AM, Demirpence E, Nicolas JC. Reporter cell lines are useful tools for monitoring biological activity of nuclear receptor ligands. Luminescence. 2001 Apr;16(2):153–8.](https://sciwheel.com/work/bibliography/1237678)

[2.    Xu Y, Huangyang P, Wang Y, Xue L, Devericks E, Nguyen HG, et al. ERα is an RNA-binding protein sustaining tumor cell survival and drug resistance. Cell. 2021 Sep 30;184(20):5215-5229.e17.](https://sciwheel.com/work/bibliography/11741011)

[3.    Tarallo R, Giurato G, Bruno G, Ravo M, Rizzo F, Salvati A, et al. The nuclear receptor ERβ engages AGO2 in regulation of gene transcription, RNA splicing and RISC loading. Genome Biol. 2017 Oct 6;18(1):189.](https://sciwheel.com/work/bibliography/7418011)

[4.    Ambrosino C, Tarallo R, Bamundo A, Cuomo D, Franci G, Nassa G, et al. Identification of a hormone-regulated dynamic nuclear actin network associated with estrogen receptor alpha in human breast cancer cell nuclei. Mol Cell Proteomics. 2010 Jun;9(6):1352–67.](https://sciwheel.com/work/bibliography/14411898)

[5.    Schneider CA, Rasband WS, Eliceiri KW. NIH Image to ImageJ: 25 years of image analysis. Nat Methods. 2012 Jul;9(7):671–5.](https://sciwheel.com/work/bibliography/222322)

[6.    Nassa G, Salvati A, Tarallo R, Gigantino V, Alexandrova E, Memoli D, et al. Inhibition of histone methyltransferase DOT1L silences ERα gene and blocks proliferation of antiestrogen-resistant breast cancer cells. Sci Adv. 2019 Feb 6;5(2):eaav5590.](https://sciwheel.com/work/bibliography/13397899)

[7.    Khodor YL, Rodriguez J, Abruzzi KC, Tang C-HA, Marr MT, Rosbash M. Nascent-seq indicates widespread cotranscriptional pre-mRNA splicing in Drosophila. Genes Dev. 2011 Dec 1;25(23):2502–12.](https://sciwheel.com/work/bibliography/1558660)

[8.    Sellitto A, Geles K, D’Agostino Y, Conte M, Alexandrova E, Rocco D, et al. Molecular and functional characterization of the somatic piwil1/pirna pathway in colorectal cancer cells. Cells. 2019 Nov 5;8(11).](https://sciwheel.com/work/bibliography/7944433)

[9.    Kidder BL. CARIP-Seq and ChIP-Seq: Methods to Identify Chromatin-Associated RNAs and Protein-DNA Interactions in Embryonic Stem Cells. J Vis Exp. 2018 May 25;(135).](https://sciwheel.com/work/bibliography/6862370)

[10.   Salvati A, Gigantino V, Nassa G, Giurato G, Alexandrova E, Rizzo F, et al. The histone methyltransferase DOT1L is a functional component of estrogen receptor alpha signaling in ovarian cancer cells. Cancers (Basel). 2019 Nov 4;11(11).](https://sciwheel.com/work/bibliography/12631494)
