# Supplementary material for: LncRNA PVT1 links estrogen receptor alpha and the polycomb repressive complex 2 in suppression of pro-apoptotic genes in hormone-responsive breast cancer
Source: Cell Death Dis. 2025 Feb 8;16(1):80. doi: 10.1038/s41419-025-07423-4 (PMC11807188; doi:10.1038/s41419-025-07423-4)
Supplement: Supplementary file 10 — Original wester blot images [file 41419_2025_7423_MOESM10_ESM.pptx]

## Slide 1
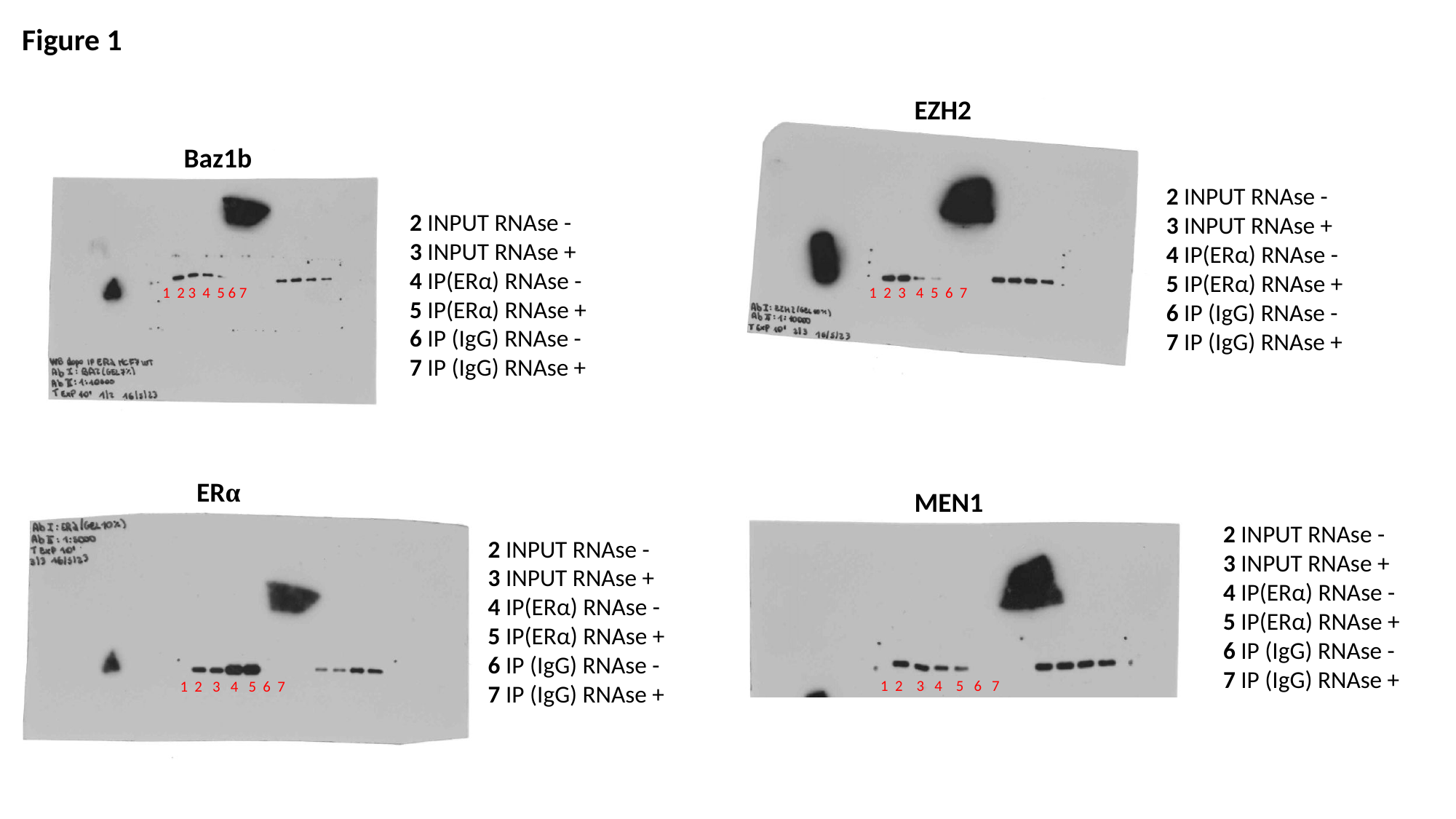

Figure 1
EZH2
Baz1b
2 INPUT RNAse -
3 INPUT RNAse +
4 IP(ERα) RNAse -
5 IP(ERα) RNAse +
6 IP (IgG) RNAse -
7 IP (IgG) RNAse +
2 INPUT RNAse -
3 INPUT RNAse +
4 IP(ERα) RNAse -
5 IP(ERα) RNAse +
6 IP (IgG) RNAse -
7 IP (IgG) RNAse +
 1 2 3 4 5 6 7
 1 2 3 4 5 6 7
ERα
MEN1
2 INPUT RNAse -
3 INPUT RNAse +
4 IP(ERα) RNAse -
5 IP(ERα) RNAse +
6 IP (IgG) RNAse -
7 IP (IgG) RNAse +
2 INPUT RNAse -
3 INPUT RNAse +
4 IP(ERα) RNAse -
5 IP(ERα) RNAse +
6 IP (IgG) RNAse -
7 IP (IgG) RNAse +
 1 2 3 4 5 6 7
 1 2 3 4 5 6 7

## Slide 2
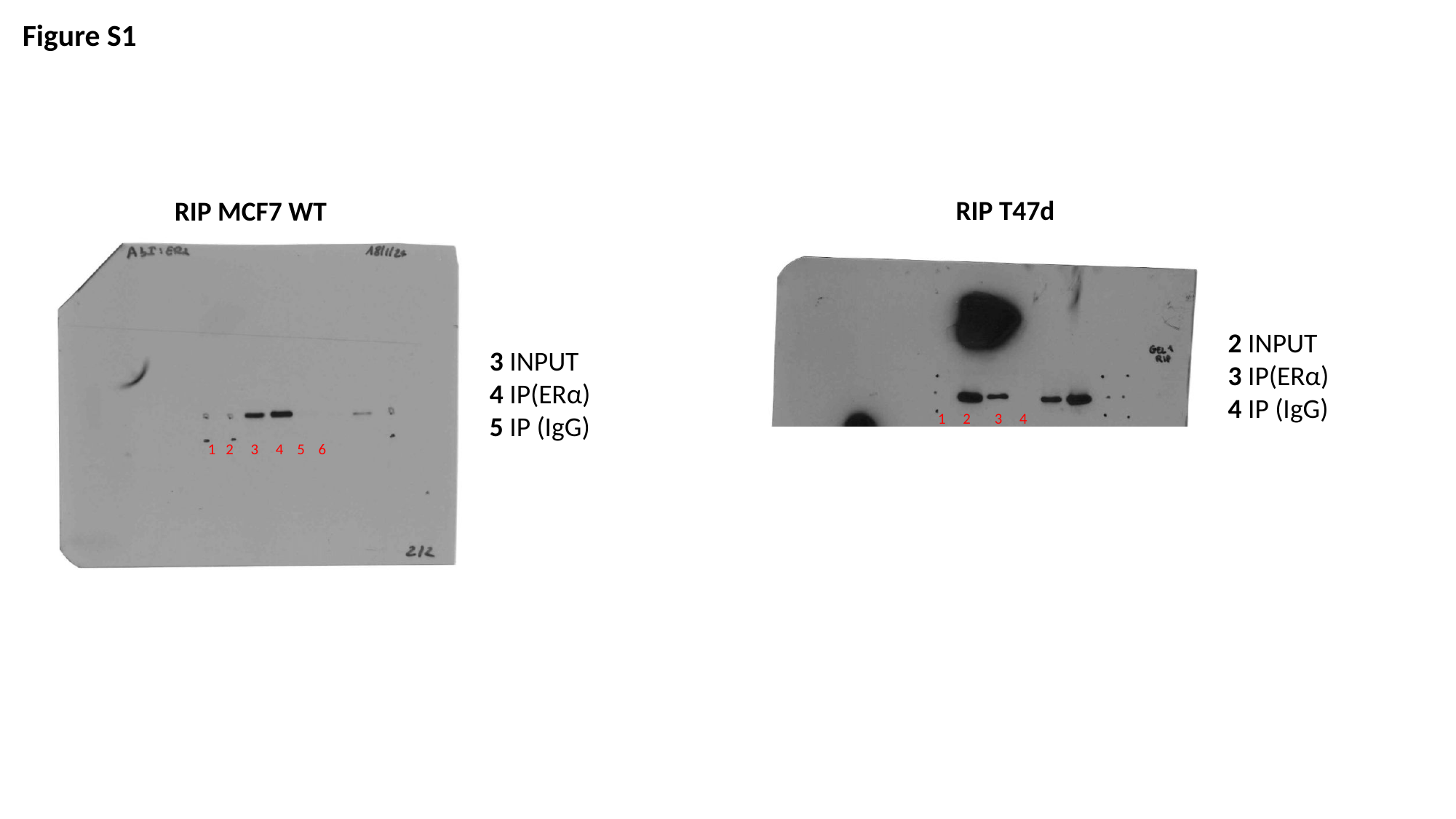

Figure S1
RIP T47d
RIP MCF7 WT
2 INPUT
3 IP(ERα)
4 IP (IgG)
3 INPUT
4 IP(ERα)
5 IP (IgG)
 1 2 3 4
 1 2 3 4 5 6

## Slide 3
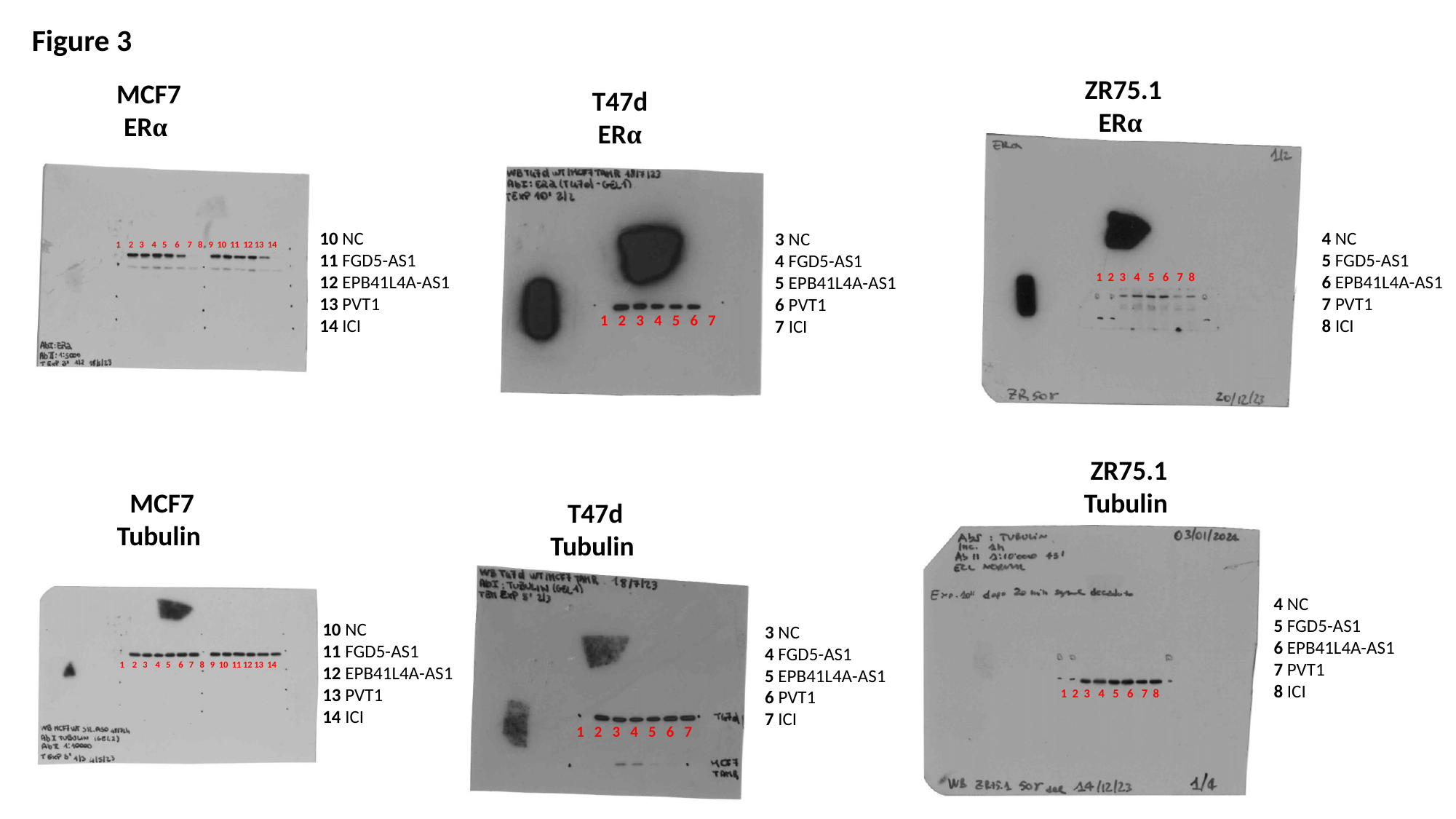

Figure 3
 ZR75.1
ERα
 MCF7
ERα
 T47d
ERα
4 NC
5 FGD5-AS1
6 EPB41L4A-AS1
7 PVT1
8 ICI
10 NC
11 FGD5-AS1
12 EPB41L4A-AS1
13 PVT1
14 ICI
3 NC
4 FGD5-AS1
5 EPB41L4A-AS1
6 PVT1
7 ICI
 1 2 3 4 5 6 7 8 9 10 11 12 13 14
 1 2 3 4 5 6 7 8
 1 2 3 4 5 6 7
 ZR75.1
Tubulin
 MCF7
Tubulin
 T47d
Tubulin
4 NC
5 FGD5-AS1
6 EPB41L4A-AS1
7 PVT1
8 ICI
10 NC
11 FGD5-AS1
12 EPB41L4A-AS1
13 PVT1
14 ICI
3 NC
4 FGD5-AS1
5 EPB41L4A-AS1
6 PVT1
7 ICI
 1 2 3 4 5 6 7 8 9 10 11 12 13 14
 1 2 3 4 5 6 7 8
 1 2 3 4 5 6 7

## Slide 4
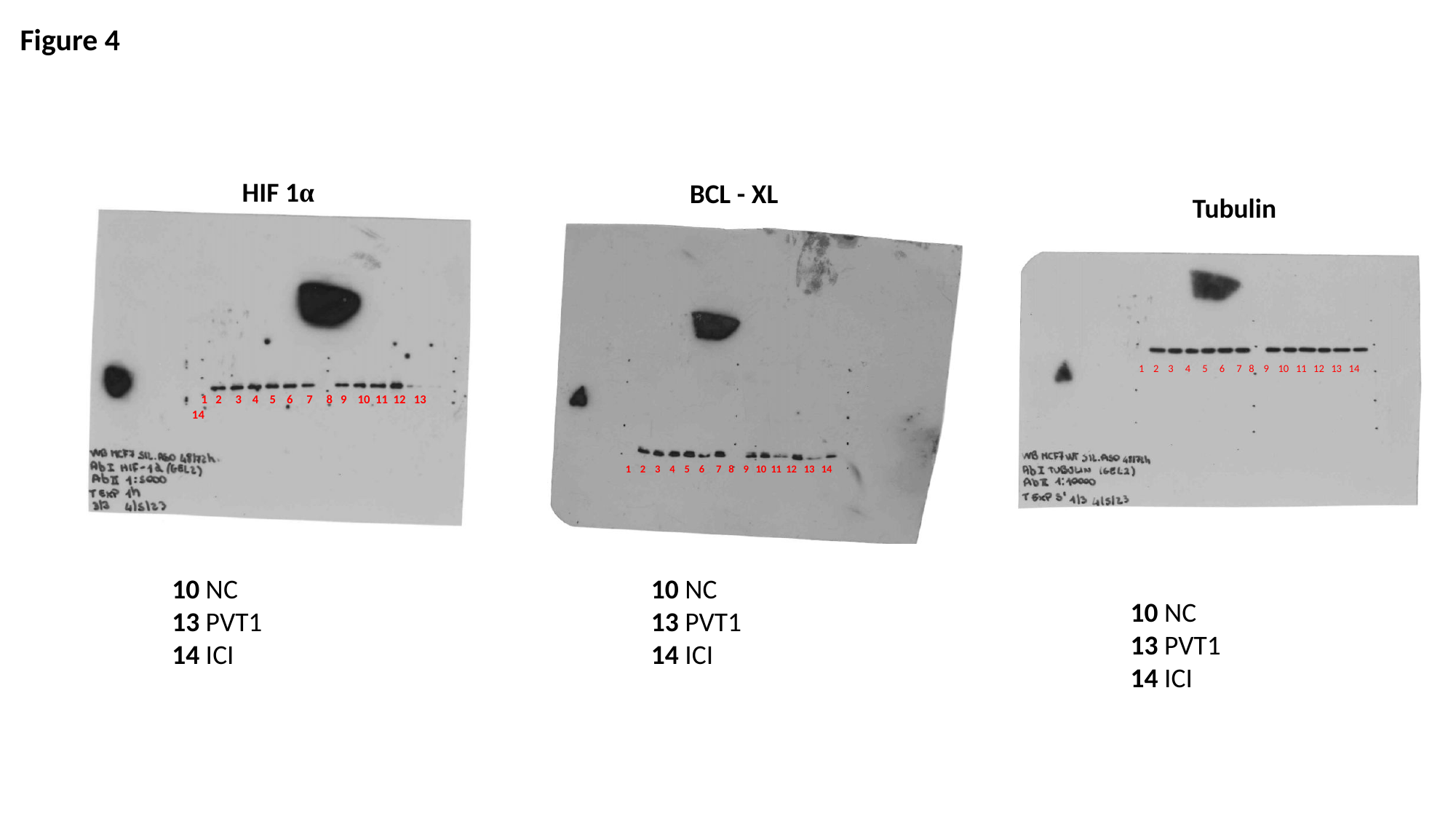

Figure 4
HIF 1α
BCL - XL
Tubulin
 1 2 3 4 5 6 7 8 9 10 11 12 13 14
 1 2 3 4 5 6 7 8 9 10 11 12 13 14
 1 2 3 4 5 6 7 8 9 10 11 12 13 14
10 NC
13 PVT1
14 ICI
10 NC
13 PVT1
14 ICI
10 NC
13 PVT1
14 ICI

## Slide 5
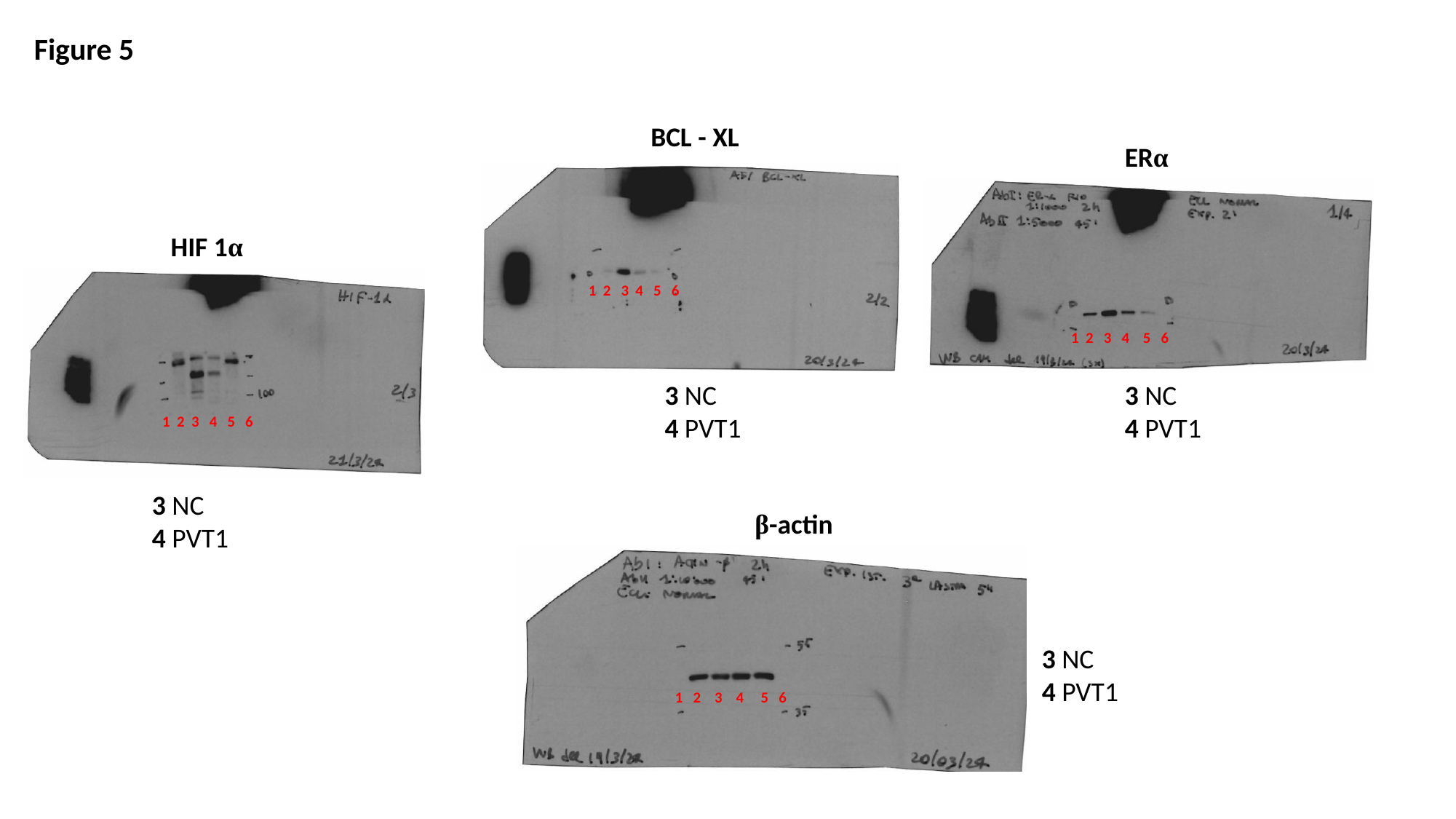

Figure 5
BCL - XL
ERα
HIF 1α
 1 2 3 4 5 6
 1 2 3 4 5 6
3 NC
4 PVT1
3 NC
4 PVT1
 1 2 3 4 5 6
3 NC
4 PVT1
β-actin
3 NC
4 PVT1
 1 2 3 4 5 6

## Slide 6
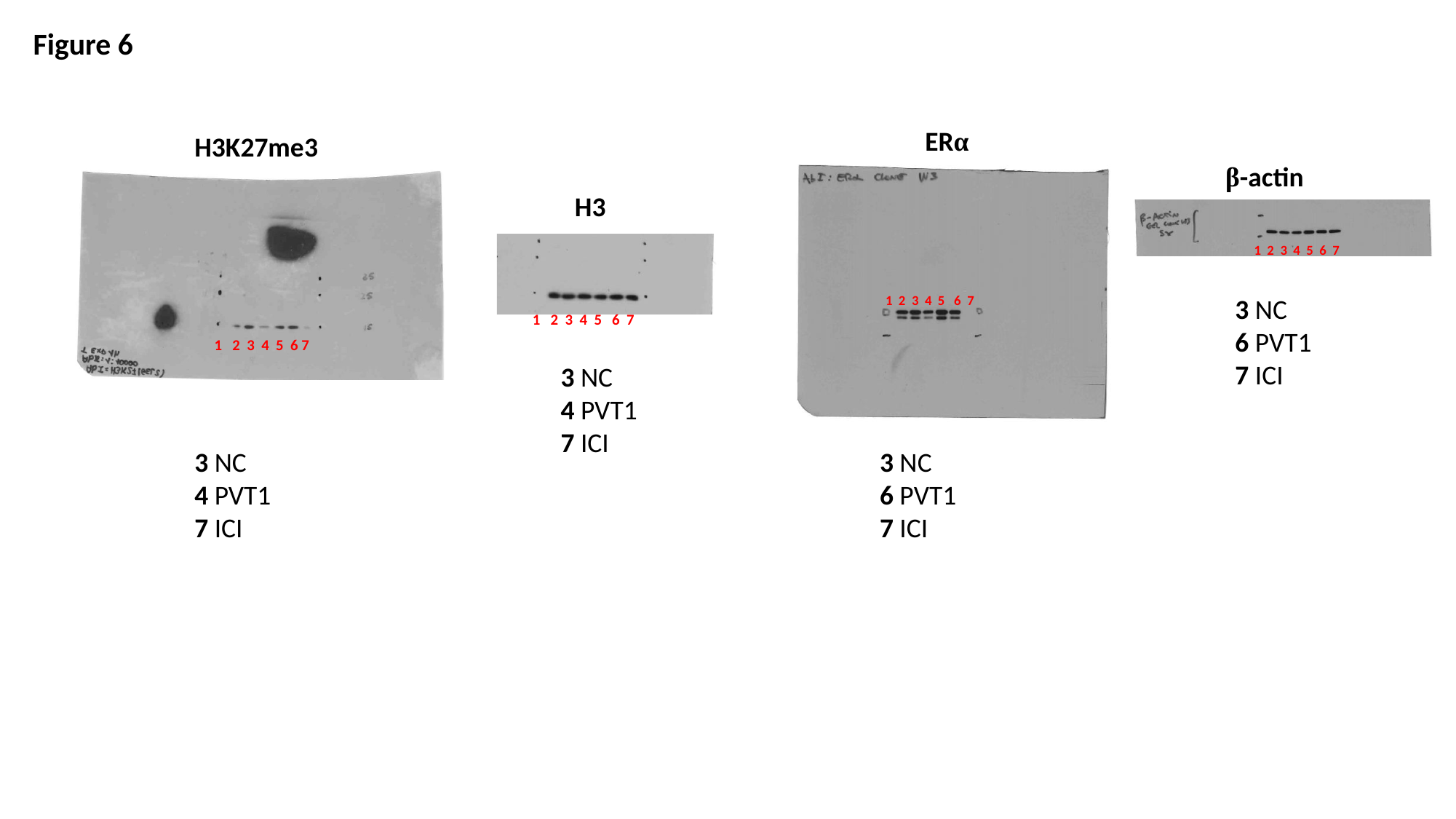

Figure 6
ERα
H3K27me3
β-actin
H3
1 2 3 4 5 6 7
1 2 3 4 5 6 7
3 NC
6 PVT1
7 ICI
1 2 3 4 5 6 7
 1 2 3 4 5 6 7
3 NC
4 PVT1
7 ICI
3 NC
4 PVT1
7 ICI
3 NC
6 PVT1
7 ICI

## Slide 7
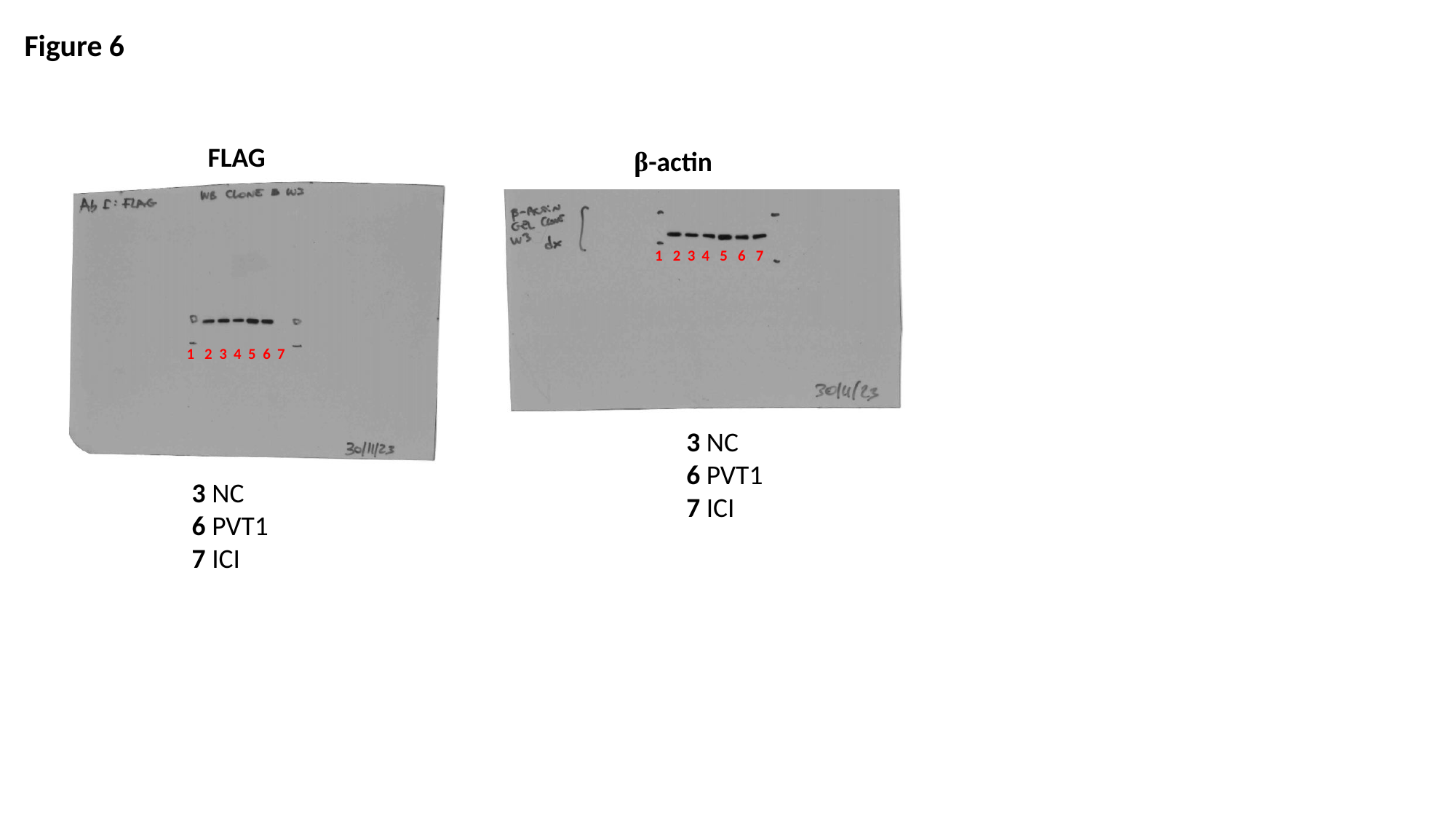

Figure 6
FLAG
β-actin
 1 2 3 4 5 6 7
 1 2 3 4 5 6 7
3 NC
6 PVT1
7 ICI
3 NC
6 PVT1
7 ICI

## Slide 8
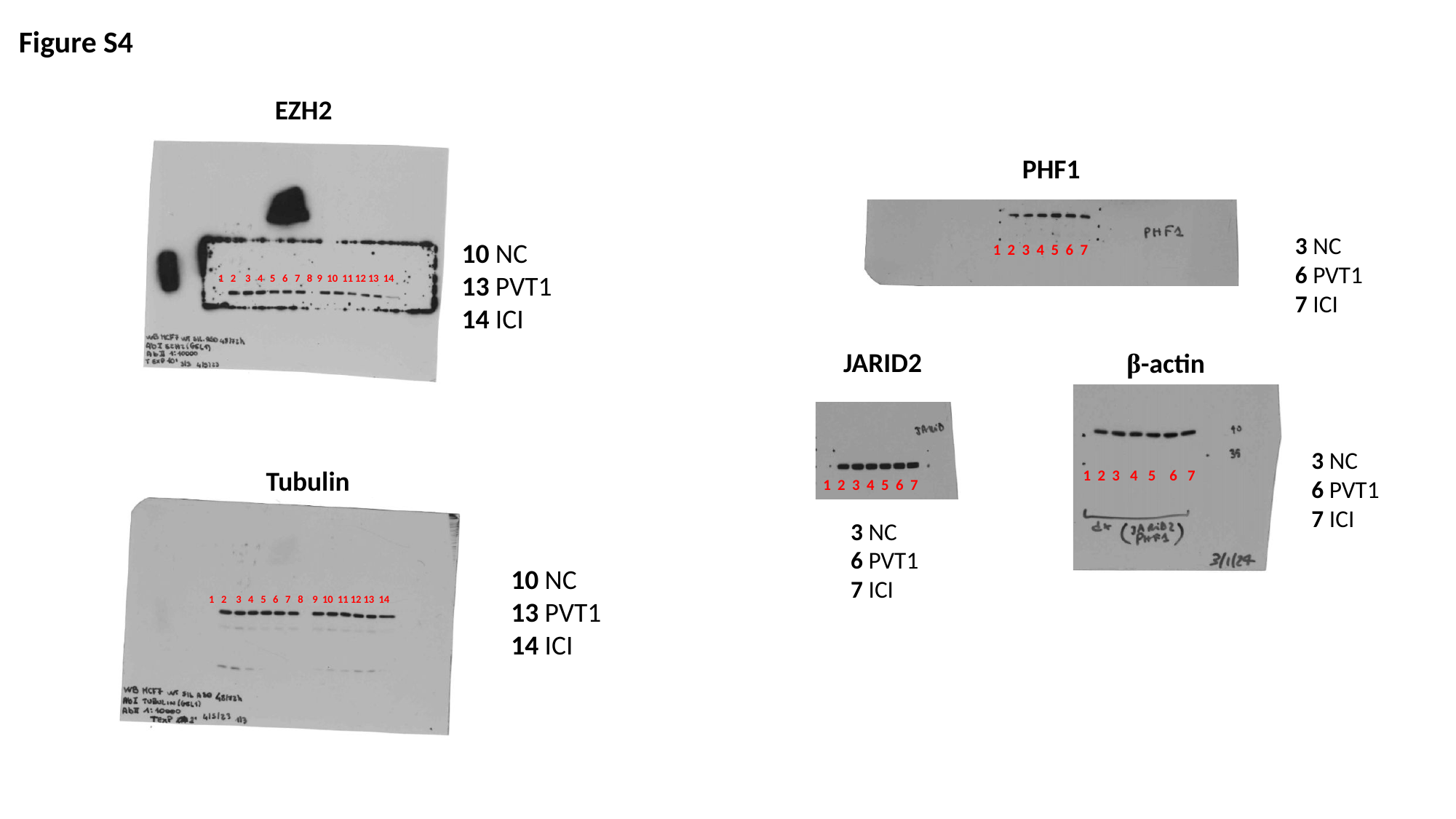

Figure S4
EZH2
PHF1
3 NC
6 PVT1
7 ICI
10 NC
13 PVT1
14 ICI
1 2 3 4 5 6 7
 1 2 3 4 5 6 7 8 9 10 11 12 13 14
JARID2
β-actin
3 NC
6 PVT1
7 ICI
Tubulin
1 2 3 4 5 6 7
1 2 3 4 5 6 7
3 NC
6 PVT1
7 ICI
10 NC
13 PVT1
14 ICI
 1 2 3 4 5 6 7 8 9 10 11 12 13 14

## Slide 9
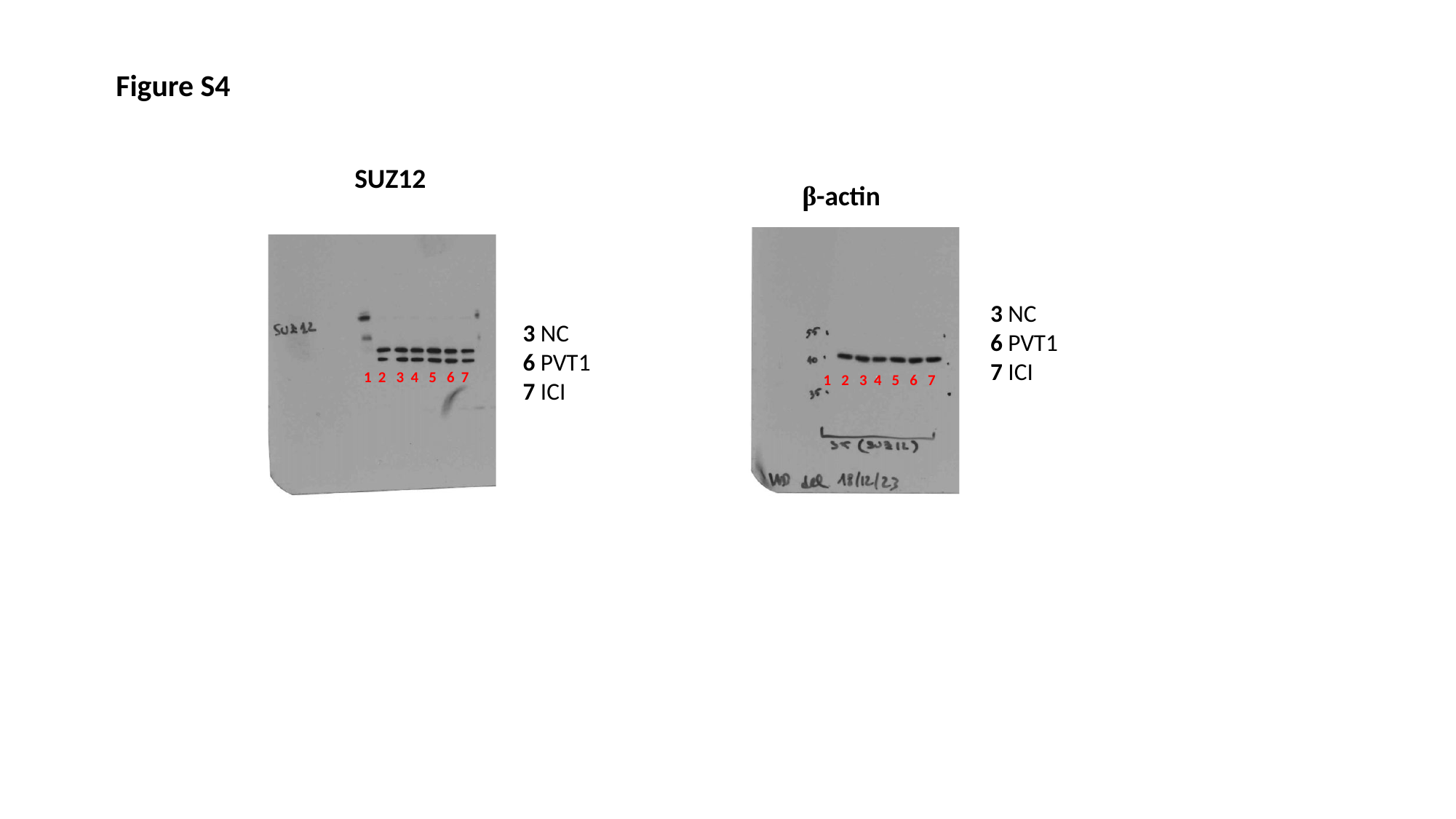

Figure S4
SUZ12
β-actin
3 NC
6 PVT1
7 ICI
3 NC
6 PVT1
7 ICI
1 2 3 4 5 6 7
1 2 3 4 5 6 7
